# Large-scale evaluation of the ability of RNA-binding proteins to activate exon inclusion

**DOI:** 10.1038/s41587-023-02014-0

**Published:** 2024-01-02

**Authors:** Jonathan C. Schmok, Manya Jain, Lena A. Street, Alex T. Tankka, Danielle Schafer, Hsuan-Lin Her, Sara Elmsaouri, Maya L. Gosztyla, Evan A. Boyle, Pratibha Jagannatha, En-Ching Luo, Ester J. Kwon, Marko Jovanovic, Gene W. Yeo

**Affiliations:** 1 Department of Cellular and Molecular Medicine, University of California San Diego, La Jolla, CA USA; 2 Sanford Stem Cell Institute Innovation Center and Stem Cell Program, University of California San Diego, La Jolla, CA USA; 3 Institute for Genomic Medicine, University of California San Diego, La Jolla, CA USA; 4 Department of Bioengineering, University of California San Diego, La Jolla, CA USA; 5 Department of Biological Sciences, Columbia University, New York, NY USA

**Keywords:** Alternative splicing, Transcriptomics, High-throughput screening, Molecular engineering

## Abstract

RNA-binding proteins (RBPs) modulate alternative splicing outcomes to determine isoform expression and cellular survival. To identify RBPs that directly drive alternative exon inclusion, we developed tethered function luciferase-based splicing reporters that provide rapid, scalable and robust readouts of exon inclusion changes and used these to evaluate 718 human RBPs. We performed enhanced cross-linking immunoprecipitation, RNA sequencing and affinity purification–mass spectrometry to investigate a subset of candidates with no prior association with splicing. Integrative analysis of these assays indicates surprising roles for TRNAU1AP, SCAF8 and RTCA in the modulation of hundreds of endogenous splicing events. We also leveraged our tethering assays and top candidates to identify potent and compact exon inclusion activation domains for splicing modulation applications. Using these identified domains, we engineered programmable fusion proteins that outperform current artificial splicing factors at manipulating inclusion of reporter and endogenous exons. This tethering approach characterizes the ability of RBPs to induce exon inclusion and yields new molecular parts for programmable splicing control.

## Abstract (web summary)

Alternative splicing is controlled with engineered fusion splicing factors.

## Main

RNA-binding proteins (RBPs) mediate myriad layers of post-transcriptional gene regulation, including alternative pre-mRNA splicing (AS)^1^. Despite the widespread importance of RBPs for cellular function, most of the more than 2,000 human proteins predicted or shown to bind RNA do not have an assigned molecular function^1,2^. AS is a prevalent and critical RNA processing step, as up to 95% of human multi-exon genes exhibit multiple splice isoforms^3^. Aberrant splicing is also widespread in disease, especially cancer^4,5^, driving proteomic imbalance and disruption of cellular homeostasis^6,7^. Among the RBPs lacking functional annotation of their RNA-binding activity are RBPs involved in AS. Systematic approaches to assign AS activity to RBPs are, thus, needed to bridge this knowledge gap.

Previous assays have employed luciferase and fluorescence-based reporter systems to identify and characterize RBPs that underscore AS. However, these have relied on global overexpression^8^ or knockdown^9,10^ of RBPs. Global perturbations of protein level are not able to separate effects caused by direct binding of RBPs from their indirect action through splicing regulatory networks. Furthermore, none of these previous studies has investigated how binding position relative to an alternatively spliced exon can modulate the effect of the RBP, even though many splicing factors can exert different effects depending on the distance and orientation (upstream or downstream of the alternative exon) of their binding position^11–14^. Reporter-based assays that recruit candidate proteins to a specific position, previously applied in studies of transcriptional effectors^15^ and modulators of RNA stability/translation^16^, are a promising avenue to address these limitations^17^.

Complementary to the important need to understand the mechanisms driving AS is the potential utility of tools for targeted modulation of splicing events. Engineered RBPs have been generated through fusion of exon activation domains to RNA-targeting PUF domains^18^ and RNA-targeting CRISPR systems^19,20^. Such technologies are in their nascent stage, reliant on exon activation domains selected from historically well-known splicing factors. A molecular toolkit of potent and compact activation domains to be implemented in maturation of these technologies remains to be established.

In this study, we developed tethered function luciferase-based splicing reporter assays to investigate and quantify the capacity of any protein sequence to directly promote exon inclusion. We used this system to systematically assess proximity-dependent modulation of exon inclusion for 718 human RBPs at two separate tethering positions and to identify potent and compact exon inclusion activation domains. Altogether, our assays serve as both a biological discovery engine that reveals factors involved in splicing and a prototyping platform that can yield molecular parts for protein engineering applications.

## Results

### Development of tethered function splicing reporter assays

**Fig. 1:**
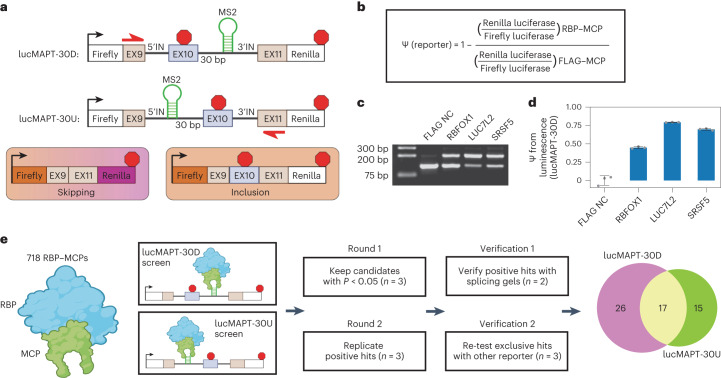
Development of tethered function assays for detecting direct induction of exon inclusion. **a**, Schematic of luciferase reporters used in the assays and resulting isoforms after cellular mRNA processing. **b**, Analysis workflow for calculating percent-spliced-in from luminescence measurements. **c**, Splicing gels of lucMAPT-30D splicing in response to co-transfection with MCP-fused positive and negative controls. Bands are generated by agarose gel electrophoresis of RT-generated cDNA amplified by minigene specific primers (shown in **a**) that amplify skipping and inclusion isoforms. **d**, Bar graph of lucMAPT-30D reporter readout as calculated from the workflow in **b** with the same conditions as **c** (mean ± s.d., *n* = 3 replicate transfections). **e**, Experimental workflow of tethering assays. The effects of recruiting 718 MCP-fused RBPs are tested in both reporter contexts. *P* value was calculated by independent two-sample one-tailed *t-*test, comparing the co-transfection of the reporter and candidates to co-transfection of the reporter and FLAG NC performed concurrently. The displayed *n* refers to biological replicates of candidate transfections. For FLAG NC transfections, *n* = 3 biological replicates for the reporter experiments and *n* = 6 for the splicing gel experiment. Venn diagram of final hits after all rounds of screening and verification. bp, base pairs.

**Extended Data Fig. 1:**
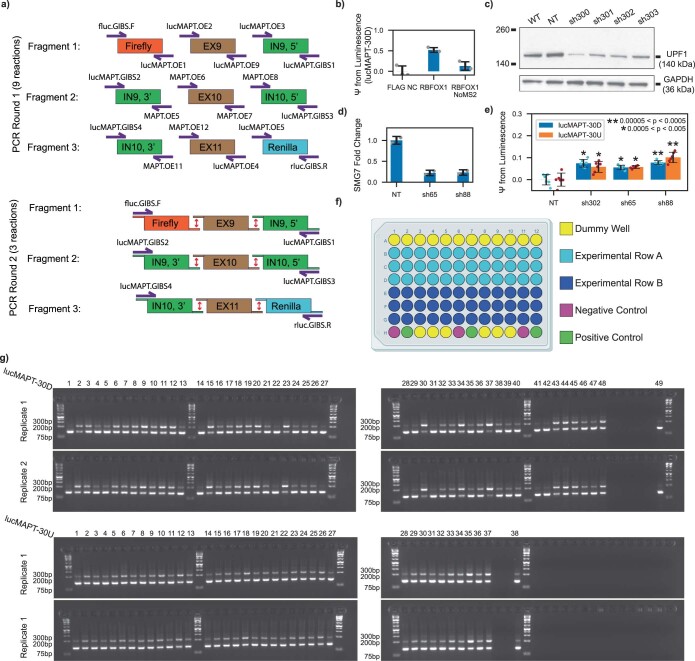
Reporter construction strategy, tethering validation, reporter layout, splicing gels. **a** Schematic of strategy used for assembling luciferase based minigene splicing reporters. **b** Bar graph of lucMAPT-30D reporter readout following co-transfection with FLAG NC, RBFOX1-MCP fusion (RBFOX1), and RBFOX1 lacking an MCP fusion (RBFOX1 NoMS2) (mean ± s.d., n = 3 replicate transfections). **c** Western blots for validation of UPF1 shRNA constructs qualitatively showing decreased UPF1 protein levels for each of four UPF1 shRNA constructs tested in HEK293T cells. **d** qPCR for validation of SMG7 shRNA constructs showing decreased SMG7 expression levels as quantified using the delta-delta Ct method in RNA extracted from MDAMB231 and MCF10A cells stably expressing the constructs (n = 2 biological replicates (1 replicate/line), n = 2 technical replicates). **e** Bar graph of reporter readouts in HEK293T cells stably expressing a non-targeting shRNA (NT), a UPF1-targeting shRNA (sh302), and two SMG7-targeting shRNAs (sh65 and sh88), co-transfected with reporter plasmids and FLAG NC (mean ± s.d., n = 6 replicate transfections). P-value is calculated by two-tailed independent two-sample *t*-test. **f** Layout of 96-well transfections used throughout the screens. **g** Agarose gels of RNA-level validation of hits from the splicing screen. All hits were tested for lucMAPT-30D (top) and lucMAPT-30U (bottom). Numbers along the top correspond to lane number in Supplementary Table 6-7. n = 2 replicate transfections. Source data

We constructed two dual-luciferase tethered AS minigene reporter systems based on the splicing event of *MAPT* *(*microtubule-associated protein tau) exon 10 (Fig. 1a and Extended Data Fig. 1a)^21^, which is predominantly excluded from the mature mRNA in HEK293T cells. The first reporter contains the MS2 hairpin 30 base pairs downstream of the 5′ splice site (lucMAPT-30D), and the second contains the MS2 hairpin 30 base pairs upstream of the 3′ splice site (lucMAPT-30U). The MS2 hairpin recruits MS2 coat protein (MCP) fused to RBP open reading frames (ORFs) to determine the effect on AS of the exon when RBPs are tethered to various positions on the RNA.

Both minigenes are flanked by a constitutively included Firefly luciferase ORF at the 5′ end and a conditionally included Renilla luciferase ORF at the 3′ end to permit inference of exon inclusion. Firefly luciferase is expressed independent of exon skipping, but inclusion of the tau exon harboring a stop codon terminates translation upstream of Renilla luciferase. We used changes in luminescence in experimental conditions to determine changes in the percent-spliced-in (ψ) of the AS exon when compared with a negative control (Fig. 1b). The AS exon is the penultimate exon, so we inserted the stop codon within 50 base pairs of the 5′ splice site to minimize sensitivity of the long isoform to nonsense-mediated decay (NMD)^22^.

To validate our assay, we co-transfected the lucMAPT-30D reporter with fusion proteins composed of known regulators of exon inclusion and MCP. For a negative control (NC), we used a construct containing an array of three FLAG epitope tags fused to MCP (FLAG NC). We compared ψ value as measured by the reporter readout to an RNA-level validation (Fig. 1c,d). Compared with FLAG NC, MCP-fused proteins LUC7L2, SRSF5 and RBFOX1 increased exon inclusion as measured by both techniques in decreasing order of intensity. To verify that effector recruitment was mediated by the MS2–MCP system, we co-transfected lucMAPT-30D with an RBFOX1 plasmid lacking the MCP fusion. This did not activate the reporter (Extended Data Fig. 1b). As we designed our reporters to minimize sensitivity to NMD, we tested the response of the reporters to NMD perturbation by testing the reporter readout in response to shRNA-mediated knockdown of UPF1, the central effector of NMD^23^, and SMG7, a non-essential NMD factor^24^ (Extended Data Fig. 1c–e). We detected a minor (<10%) increase in long isoform abundance after NMD perturbation, indicating that the early stop codon-containing long isoform is, to some degree, sensitive to NMD. For the purposes of our studies, where the NMD environment is consistent and candidates are recruited specifically to pre-mRNA by MS2-containing introns, we deemed it acceptable. Based on these validations, we moved forward with these reporters to screen our RBP–MCP library.

### Tethering assays identify RBPs that induce exon inclusion

We evaluated 718 RBP ORFs fused to MCP for their ability to induce exon inclusion (Supplementary Table 1). Our laboratory previously developed the RBP–MCP library from subcloning of putative RBP ORFs^16^. We performed two arrayed co-transfection screens with candidate RBPs in HEK293T cells, one with lucMAPT-30D and one with lucMAPT-30U (Fig. 1e, left). We analyzed all ORFs in triplicate and compared with negative controls (FLAG NC) and positive controls (RBFOX1-MCP for lucMAPT-30D and SRSF5-MCP for lucMAPT-30U) on the same plate (Extended Data Fig. 1f). Because our analysis focused on ψ increases exclusively, we measured statistical significance when compared with the negative control by one-tailed independent two-sample *t*-test.

We moved forward with candidates that increased ψ significantly (*P* < 0.05; Supplementary Tables 2 and 3) and verified them with further rounds of screening (Fig. 1e, middle). First, we replicated the reporter results of all selected candidates and moved forward with those that again increased ψ significantly (*P* < 0.05; Supplementary Tables 4 and 5). We then verified that all positive hits induced exon inclusion of the reporter at the RNA level through agarose gel electrophoresis of amplified cDNA following the same transfection conditions (Extended Data Fig. 1g and Supplementary Tables 6 and 7). ψ was estimated by calculating the intensity ratio of the inclusion band to the skipping band in duplicate and comparing against control conditions distributed throughout the gel. We calculated *P* value by one-tailed independent two-sample *t*-test, and hits with Bonferroni-corrected *P* < 0.05 were kept. Finally, remaining hits that exclusively activated one of the two reporters were evaluated one more time with the opposite reporter in case they were missed by the initial screen (Supplementary Tables 8 and 9). After these rounds of screening, 26 hits were detected that exclusively activated lucMAPT-30D; 15 hits were detected that exclusively activated lucMAPT-30U; and 17 hits were detected that activated both reporters (Supplementary Table 10 and Fig. 1e, right).

**Fig. 2:**
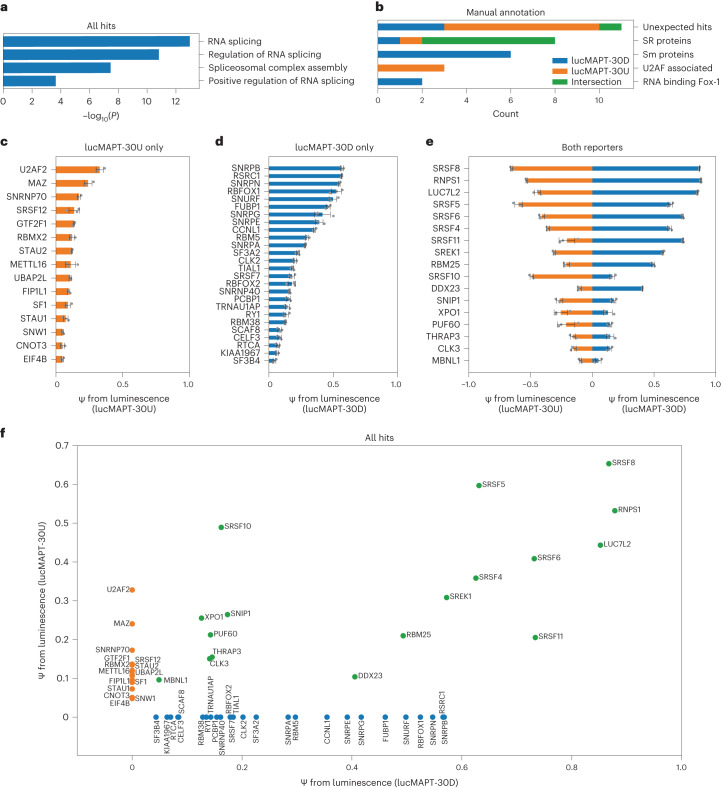
Tethering assays identify RBPs that induce exon inclusion. **a**, Bar graph displaying GO analysis of all hits emerging from the screen. The top four mostly significantly enriched biological processes are displayed. Background for analysis is the complete list of genes used in the screens. Unadjusted *P* value was calculated using Metascape^56^ based on the accumulative hypergeometric distribution. **b**, Bar graph displaying manual annotation of protein families displaying reporter preferences in the screen and reporter preferences of unexpected hits. **c**–**e**, Bar graphs displaying reporter readout after screen and validation for RBP–MCP fusions of all final hits passing the lucMAPT-30U screen only (**c**), the lucMAPT-30D screen only (**d**) and both screens (**e**) (mean ± s.d., *n* = 3 replicate transfections). **f**, Scatter plot comparing activation of both reporters of all hits emerging from the screen. Hits that passed only for a single reporter are placed along the axis for the other reporter. Hits that passed the lucMAPT-30U screen only are displayed as orange markers; hits that passed the lucMAPT-30D screen only are displayed as blue markers; and hits that passed both screens are displayed as green markers.

We investigated the biology underlying the candidates detected from our screens. To verify that our assays robustly captured known regulators of AS, we performed Gene Ontology (GO) analysis on the full list of final hits. When compared with a background of the complete tethering library, GO analysis showed strong enrichment of RNA splicing-associated terms (Fig. 2a). As AS occurs in the nucleus, we investigated the subcellular localization of the candidates. We referenced the COMPARTMENTS subcellular localization database, which integrates evidence from text mining, high-throughput screens, literature and prediction methods, and extracted the nuclear localization confidence score for each candidate^25^. All candidates, save two, have a nuclear confidence score of 4/5 or greater (Supplementary Table 10). The two candidates that scored lower than 4/5 were STAU1 and EIF4B. STAU1, which scored 2.68/5, has previously been linked to splicing regulation^26,27^. EIF4B, which scored 3.82/5, initiates translation in the cytoplasm by binding RNA substrates and recruiting ribosomes. We hypothesize that this mechanism could drive a false positive when artificially driven to nuclear pre-mRNA in our tethering system, as the mechanism of spliceosome recruitment is similar. Nevertheless, a potentially nuclear role of EIF4B in splicing regulation merits future investigation. Altogether, the candidates determined by our screen are enriched for known regulators of mRNA splicing and are largely localized to the nucleus.

We also detected differences in the types of RBPs identified by each screen (Fig. 2b). Both RBFOX1 and RBFOX2 exclusively activated the reporter when tethered downstream (lucMAPT-30D), consistent with the known effect of these proteins primarily causing exon inclusion when bound downstream of alternatively spliced exons^11,28^. Three proteins associated with 3′ splice site recognition exclusively activated the upstream tethering reporter (lucMAPT-30U): U2AF2 (the large subunit of the U2 auxiliary factor), SF1 and SNW1 (refs. ^29,30^). The RBPs tested from the Sm family (SNRPB, SNRPN, SNURF, SNRPG, SNRPE and SNRPA) exclusively and potently activated the downstream tethering reporter, despite the Sm ring being found in spliceosomal subunits that form at either end of the splicing junction^31^. The SR family of splicing factors was primarily represented at the intersection of both screens (SRSF8, SRSF5, SRSF6, SRSF4, SRSF11 and SRSF10); however, SRSF7 exclusively activated the downstream tethering reporter, and SRSF12 exclusively activated the upstream tethering reporter.

As we were especially interested in candidates that have not previously been associated with AS regulation, we first determined candidates that were not annotated with splicing-associated GO terms and have not been specifically referenced in the literature as potential splicing factors and deemed them ‘unexpected hits’. Most unexpected hits exclusively activated the upstream tethering reporter (UBAP2L, STAU2, EIF4B, CNOT3, MAZ, GTF2F1 and FIP1L1), which was uncommon for known splice modulatory factors. We detected three unexpected hits as exclusive activators of the downstream tethering reporter (TRNAU1AP, SCAF8 and RTCA) and one as an activator of both reporters (XPO1). Next, we searched for the unexpected hits on the spliceosome database (SpliceosomeDB) to determine if previous proteomics efforts have identified them as interactors with components of the spliceosome in humans^32^. This search yielded such evidence for SCAF8, CNOT3 and FIP1L1. SCAF8 has been detected in a supraspliceosome complex in vivo assembled from HeLa cell extract^33^ and after immunoprecipitation of CDC5L in HeLa cells^34^. CNOT3 has been detected after immunoprecipitation of SRRM1 in HeLa extract^35^. FIP1L1 has been detected after isolation of mixed spliceosome complexes assembled in vitro from the extracts of WERI-1 retinoblastoma cells^36^ and HeLa cells^37^. Finally, we also noted that XPO1 has a known, albeit indirect, role in mRNA splicing. XPO1 is a nuclear export receptor that shuttles the immature small nuclear RNAs (snRNAs) of the spliceosome to the cytoplasm for maturation^38^. Despite the preliminary evidence linking a subset of the unexpected hits to mRNA splicing, the landscape of splicing events regulated by any of the unexpected hits has not currently been characterized in any biological system.

We binned hits into categories depending on whether they activated the downstream reporter only, activated the upstream reporter only or activated both reporters. Binned RBPs display effect size patterns associated with their categories (Fig. 2c–f). For the RBPs that activated both reporters, ψ for the two reporters is correlated. A population exists among the RBPs that activated both reporters with high strength, which includes the strongest overall hit, SRSF8. SRSF8 activated the highest ψ with the upstream tethering reporter and the second highest ψ for the downstream tethering reporter behind RNPS1. The downstream-only hits generally exhibited stronger activation than upstream-only hits. These categories of hits display trends in effect size; however, the variance within each category highlights the diversity of mechanisms by which RBPs influence AS by proximity.

**Extended Data Fig. 2:**
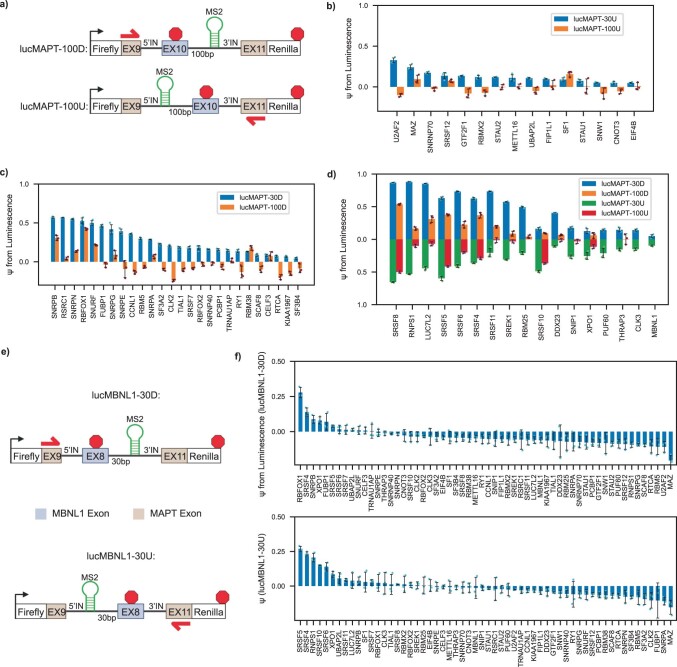
Survey of screen hits with complementary reporters. **a** Schematic of luciferase reporters for tethering 100 base pairs away from the splice site. **b** Clustered bar graph of upstream tethering only hits from the screen comparing results from the original screen (lucMAPT-30U) to results from co-transfection of the RBP-MCP fusions and lucMAPT-100U (mean ± s.d., n = 3 replicate transfections). **c** Clustered bar graph of downstream tethering only hits from the screen comparing results from the original screen (lucMAPT-30D) to results from co-transfection of the RBP-MCP fusions and lucMAPT-100D (mean ± s.d., n = 3 replicate transfections). **d** Clustered bar graph of hits that activated both reporters from the screen comparing results from the original screens (lucMAPT-30D, lucMAPT-30U) to results from co-transfection of the RBP-MCP fusions and the long-distance reporters (lucMAPT-100D and lucMAPT-100U) (mean ± s.d., n = 3 replicate transfections). Results where hits displayed a mean ψ from luminescence < 0 are omitted for clarity. **e** Schematic of lucMBNL1 reporters used as orthogonal exon inclusion reporters. **f** Bar graphs of reporter readout from co-transfection of all hits from the original screens with lucMBNL1-30D and lucMBNL1-30U (mean ± s.d., n = 3 replicate transfections).

We also tested our final collection of hits with orthogonal exon inclusion reporters. We screened our hits using lucMAPT reporters containing tethering sites 100 base pairs distal to the splice site instead of 30 base pairs (Extended Data Fig. 2a). Almost all hits exhibited reduced activity at the increased distance, but proximity dependence varied by RBP (Extended Data Fig. 2b–d and Supplementary Tables 11 and 12). Finally, we tested all hits with another exon inclusion reporter based around *MBNL1* exon 8 (lucMBNL1; Extended Data Fig. 2e). Although positive control SRSF5 successfully induced exon inclusion, the baseline inclusion rate was perturbed by a small subset of hits, implying some context dependence of proximity-dependent splicing activity of the tested RBPs (Supplementary Tables 13 and 14 and Extended Data Fig. 2f). Nevertheless, the lucMAPT screens provide one valid context, and we continued forward with their findings with the knowledge that we are capturing effects within it.

**Extended Data Fig. 3:**
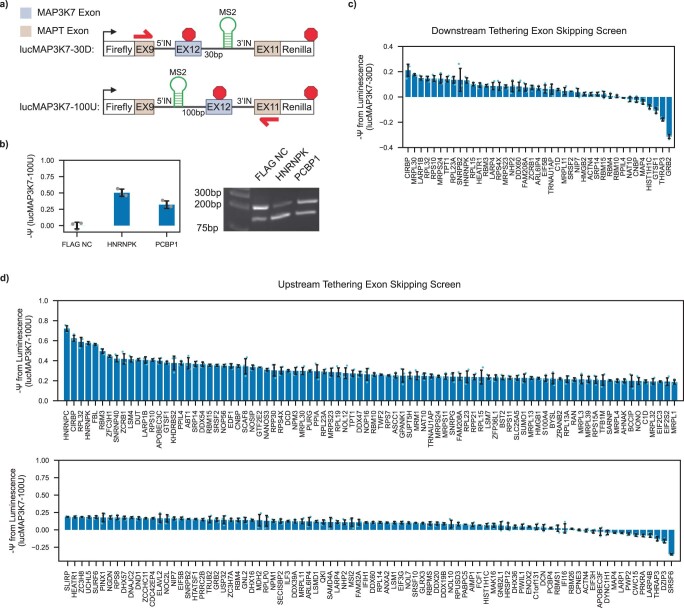
Exon skipping screen. **a** Schematic of luciferase reporters for skipping readout. **b** lucMAP3K7-100U splicing in response to co-transfection with MCP-fused positive and negative controls. (left) Bar graph of lucMAP3K7 reporter readout (mean ± s.d., n = 3 replicate transfections). (right) Agarose gel electrophoresis of RT-generated cDNA amplified by minigene specific primers (shown in panel a) that amplify skipping and inclusion isoforms. **c** Bar graph of lucMAP3K7-30D reporter readout when co-transfected with RBP-MCP fusions from the library. (mean ± s.d., n = 3 replicate transfections). **d** Bar graph of lucMAP3K7-100U reporter readout when co-transfected with RBP-MCP fusions from the library (mean ± s.d., n = 3 replicate transfections).

Initially, we also investigated a complementary approach to identify RBPs that induce exon skipping. We constructed a reporter using the same framework around *MAP3K7* exon 12, which is primarily included in HEK293T cells (Extended Data Fig. 3a). We validated the response of the *MAP3K7* reporter to HNRNPK and PCBP1, known activators of exon skipping, using the reporter readout and RNA-level validation when tethered 100 base pairs upstream of the AS exon (Extended Data Fig. 3b). Twenty-two of 44 RBPs induced exon skipping when tethered 30 base pairs downstream of the AS exon, and 154 of 194 induced exon skipping when tethered 100 base pairs upstream of the AS exon (Extended Data Fig. 3c,d and Supplementary Tables 15 and 16). The high proportion of hits suggests that recruitment of many proteins may simply act to sterically prevent spliceosome recognition; thus, we stopped the skipping screen here and constrained this study to focus on exon inclusion, a more specific molecular task.

### Splicing events are modulated by unexpected hits

We followed up the screen with endogenous characterization of four hits from the screen, which, to this point, have no established role in AS regulation: STAU2, SCAF8, RTCA and TRNAU1AP. STAU2 is an important protein in neuronal mRNA localization^39^ that shares 59.9% similarity with paralogue STAU1: a multi-functional RBP with implications for oncogenesis and neurodegeneration^26,40^. SCAF8 was previously characterized for roles in selection of distal poly(A) sites and transcriptional elongation, and a selection of genes in the same family are known or predicted to be involved in AS, including SCAF1, SCAF4 and SCAF11 (ref. ^41^). Although SCAF8 was detected in two previous spliceosomal proteomics experiments, the significance of this finding has not been further investigated^33,34^. RTCA has been previously characterized for its role in RNA metabolism by catalyzing the conversion of the 3′ phosphate of RNA substrates to a 2′,3′-cyclic phosphodiester^42^. TRNAU1AP is a poorly characterized protein predicted to play a role in selenocysteine (Sec) biosynthesis and incorporation into selenoproteins^43^. The four unexpected candidates selected vary widely in structure and currently defined function. To assess whether these are bona fide splicing factors, we applied functional genomics approaches to investigate the activity of the unexpected candidates in cells.

**Extended Data Fig. 4:**
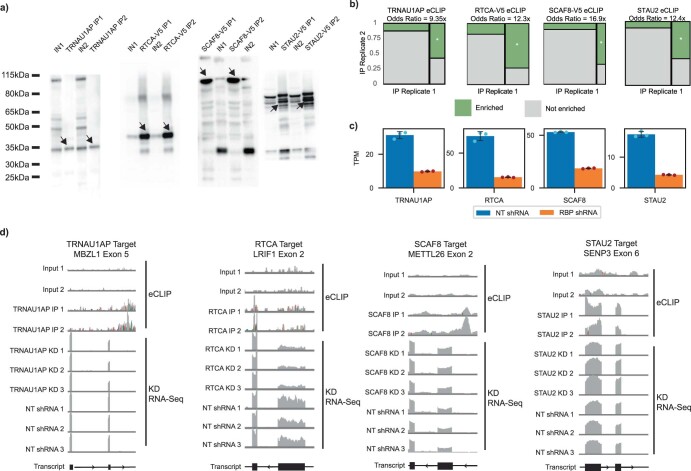
Quality control of eCLIP and shRNA knockdown followed by RNA-seq. **a** Western blots of cold gels from eCLIP protocol for TRNAU1AP, SCAF8, STAU2 and RTCA. Size-matched input and immunoprecipitation conditions are compared. n = 2 independent samples, with size-matched input and IP conditions extracted from both. **b** Mosaic plots from Skipper showing concordance between eCLIP replicates. Odds ratios and significance from Fisher’s exact test. **c** TPM of unexpected hits following shRNA knockdown as measured from aligned RNA-seq data. (mean ± s.d., n = 3 replicate knockdowns). **d** IGV browser tracks showing coverage of RBP eCLIP signal relative to sized-matched input and the RBP KD RNA-Seq signal relative to non-targeting shRNA. From left to right: comparison of TRNAU1AP eCLIP and KD RNA-Seq signal near MBZL Exon 5, comparison of RTCA eCLIP and KD RNA-Seq signal near LRIF Exon 2, comparison of SCAF8 eCLIP and KD RNA-Seq signal near METTL26 Exon 2, comparison of STAU2 eCLIP and KD RNA-Seq signal near SENP3 Exon 6.

We first interrogated endogenous RNA targets and transcriptome-wide binding sites of the unexpected candidates using enhanced cross-linking immunoprecipitation (CLIP) followed by sequencing (eCLIP)^44^ in HEK293T cells. For TRNAU1AP, we performed eCLIP using an immunoprecipitation (IP)-grade specific antibody^45^. For the other unexpected hits that did not have IP-grade antibodies available, we expressed V5-tagged ORFs and performed eCLIP with a validated V5 antibody. We successfully completed IP for all replicates (Extended Data Fig. 4a). We retrieved enriched windows using the Skipper pipeline^46^ and found them to be reproducible across two independent replicates each for all eCLIP experiments (concordance odds ratio (OR) > 9× for all experiments; Extended Data Fig. 4b).

**Fig. 3:**
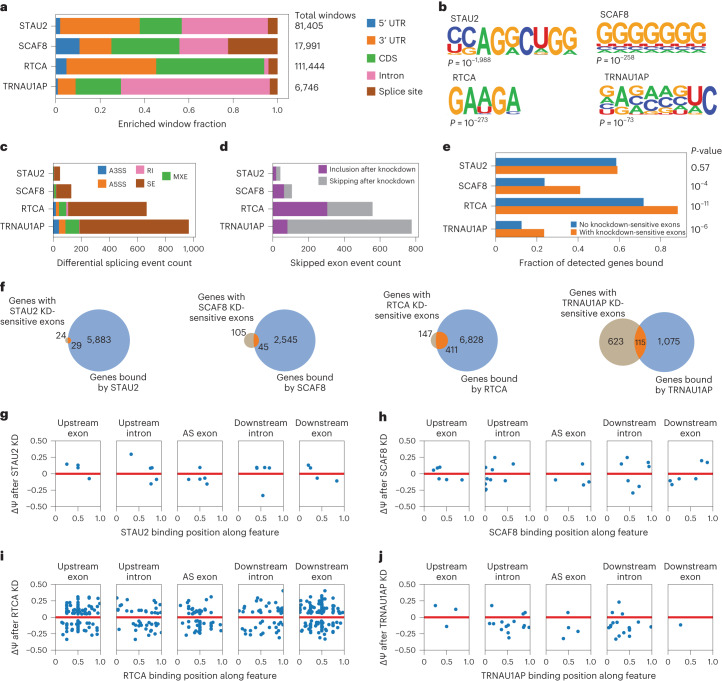
Integrated analysis of eCLIP and knockdown RNA-seq reveals splicing events modulated by unexpected hits from the tethering assay. **a**, Stacked bar graph displaying distribution of RBPs of unexpected hits identified by eCLIP and Skipper analysis (*n* = 2 for IP and sized-matched input). **b**, The most significantly enriched RNA binding motifs as identified by HOMER^57^ analysis of eCLIP signal of unexpected hits along with corresponding *P* value, as calculated by the HOMER algorithm using default settings. **c**, Stacked bar graph of differentially spliced events after shRNA-mediated knockdown of unexpected hits as identified by rMATS analysis of RNA-seq data. Differentially spliced events are called as those with an inclusion level difference >0.05 and a multiple hypothesis-adjusted *P* < 0.05 as calculated by the likelihood ratio test. **d**, Stacked bar graph of SE events after shRNA-mediated knockdown of unexpected hits as identified by rMATS analysis of RNA-seq data. Differentially spliced events are called as those with an inclusion level difference >0.05 and a multiple hypothesis-adjusted *P* < 0.05 as calculated by likelihood ratio test. Events called as ‘skipping after knockdown’ are differentially spliced events with IncLevelKD − IncLevelNT < 0. Events called as ‘inclusion after knockdown’ are differentially spliced events with IncLevelKD − IncLevelNT > 0. **e**, Bar graph showing the fraction of genes containing significantly enriched windows after eCLIP of unexpected hits, binned into genes containing corresponding knockdown-sensitive skipped exons events and those without. *P* value was calculated by one-tailed Fisher exact test. **f**, Venn diagrams displaying the number of genes containing differentially spliced skipped exon events after unexpected hit knockdown and the number of genes containing significantly enriched windows after the corresponding eCLIP. **g**–**j**, Scatter plots examining genes containing unexpected hit knockdown-sensitive skipped exon events and corresponding significantly enriched binding windows. Binding positions were stratified by feature relative to the skipped exon. When multiple binding windows were identified on the same feature relative to an exon, the median binding window is displayed. **g**, STAU2. **h**, SCAF8. **i**, RTCA. **j**, TRNAU1AP. KD, knockdown.

To determine the RNA region preferences of the candidate proteins, we examined the region annotation of all reproducible enriched windows from the eCLIP signals (Fig. 3a). STAU2 reproducible enriched windows were represented most frequently in intronic regions and the 3′ untranslated region (UTR) (also consistent with its known role in RNA localization). The reproducible enriched windows of SCAF8 were frequently near splice junctions, indicative of splicing regulation, with a relatively even distribution of regions otherwise. RTCA displayed widespread binding (>100,000 reproducible enriched binding windows), with a robust preference for coding sequence and 3′ UTR (consistent with its role in 3′ RNA processing) binding and a strong under-enrichment of intronic binding when compared with the other candidates. TRNAU1AP binding sites showed a stark preference for intronic binding, resembling the binding patterns of some well-described splicing factors, such as RBFOX2 and HNRNPC^28^. From region binding alone, we saw patterns in SCAF8 and TRNAU1AP binding that are reflective of known splicing factor binding and patterns among the other candidates that indicate that, although the proteins may be able to modulate splicing, they play major roles in other RNA processing steps as well.

Next, we performed motif analysis on the reproducible enriched windows in the eCLIP signal for each of the unexpected hits (Fig. 3b). The top motif for RTCA is part of the known exonic splicing enhancer hexamer sequence 5′-GAAGAA-3′ (ref. ^47^). The top motif for SCAF8 is a poly(G) run, associated with AS regulation^48,49^. Overall, examination of the top motif contained within each of the eCLIP signals revealed that RTCA and SCAF8 bind to signals associated with splicing regulation.

To investigate whether these RBPs modulate AS of endogenous RNA, we performed shRNA-mediated knockdown followed by RNA sequencing (RNA-seq) analysis in HEK293T cells with shRNAs specific to these proteins. Knockdowns of all targets were successful, with knockdown of at least 50% as measured by transcripts per million (TPM) (Extended Data Fig. 4c). We examined the differential AS events after knockdown and detected differentially spliced events for all knockdowns (Fig. 3c). To simplify characterization, we performed further analysis on differentially spliced events of the skipped exon (SE) category. At least 30 differential SE events were driven by the knockdown of each of these candidates. For RTCA and TRNAU1AP, more than 500 differentially spliced events were detected. We determined the direction of splicing change for each differentially spliced SE event (Fig. 3d). As the initial screens were designed to detect RBPs with the potential to induce exon inclusion, we expected to observe splicing events with increased skipping upon knockdown. We observe this trend for TRNAU1AP, indicating that TRNAU1AP is endogenously driving exon inclusion, matching our prediction from the screens. The other candidates did not display the same trend. Nevertheless, they cannot be eliminated as direct drivers of exon inclusion at this stage, because final AS outcome also captures participation of the unexpected hits in upstream pathways and competitive effects with other splicing factors^50^. The data here indicate that the candidates each play roles in AS regulation of some events, with TRNAU1AP and RTCA modulating many SE events.

To nominate AS exons that could be regulated by direct binding, we integrated findings from eCLIP and RNA-seq. We found that genes containing knockdown-sensitive exons are bound at a significantly higher rate than genes lacking knockdown-sensitive exons by SCAF8, RTCA and TRNAU1AP but not by STAU2 (Fig. 3e,f). Although the count of genes containing knockdown-sensitive SE events is low for STAU2 in comparison to the count of genes bound, the events in which there is overlap could be directly driven by binding; however, this appears to be a more specific than widespread phenomenon, at least in HEK293T cells. RTCA binds to most genes containing knockdown-sensitive SE events, indicating that the binding of RTCA directly drives many splicing changes. TRNAU1AP and SCAF8 both bind a substantial portion of genes with knockdown-sensitive SE events. Splicing modulation of these events may be directly driven by this binding. Some of the non-bound differential splicing events could by driven by their roles in pathways upstream of splicing outcome or could be bound at levels below the detection sensitivity of eCLIP. Altogether, RTCA, SCAF8 and TRNAU1AP appear to directly regulate many SE events through binding, whereas STAU2 appears to do this in a more limited capacity.

To investigate individual cases of our candidates directly driving AS modulation through position-dependent binding, we generated maps of knockdown-sensitive splicing events containing nearby binding signal. We found instances of candidate RBP binding to knockdown-sensitive exons as well as flanking introns and exons and plotted the center of the reproducible enriched binding windows across these features against the change in exon inclusion level after knockdown (Fig. 3g–j). At the few sites with STAU2 binding and STAU2 knockdown-sensitive splicing, no clear pattern emerges, indicating that direct STAU2-mediated splicing change is not a widespread and generalized phenomenon (Fig. 3g). Binding of SCAF8 is distributed throughout AS exons as well as the flanking introns and exons (Fig. 3h). SCAF8 frequently binds at the upstream 5′ splice site of exons that are skipped after knockdown. RTCA binding is prevalent in AS exons, flanking introns and flanking exons, with most prevalent binding in the flanking exons (Fig. 3i). We detected knockdown-sensitive splicing changes in both directions with nearby RTCA binding. TRNAU1AP commonly binds the flanking introns of exons that are skipped after knockdown, with a cluster present at the downstream 5′ splice site, implying that TRNAU1AP binds downstream of alternatively spliced exons and induces exon inclusion (Fig. 3j). This matches the position-dependent effect captured in the initial screen. To visualize specific instances of direct splicing regulation, we generated genome tracks of sample targets with knockdown-sensitive differential splicing and nearby eCLIP signal for TRNAU1AP, RTCA, SCAF8 and STAU2 (Extended Data Fig. 4d). In summary, we used integrated analysis of eCLIP and knockdown RNA-seq to identify instances of direct SE modulation by binding of STAU2, SCAF8, RTCA and TRNAU1AP with SCAF8, RTCA and TRNAU1AP displaying interesting position-dependent modulatory trends.

### Splicing protein enrichment in pulldown of unexpected hits

**Fig. 4:**
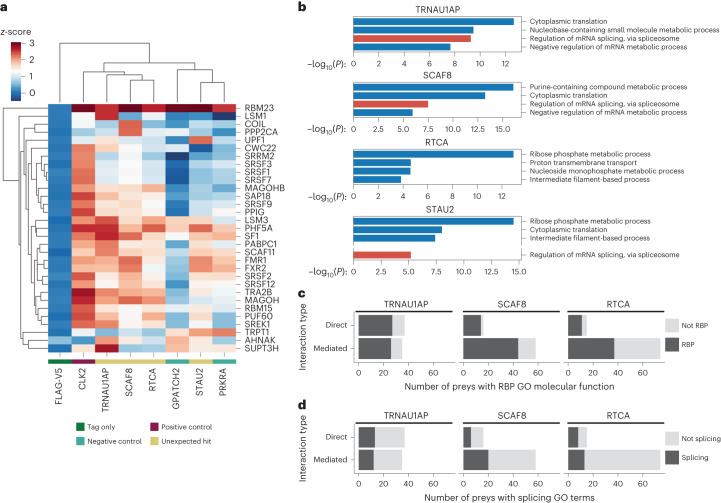
AP–MS identifies splicing-associated proteins after pulldown of candidate proteins. **a**, Hierarchically clustered heat map displaying *z*-scores from AP–MS of annotated splicing-associated preys that were detected with *z*-score > 2 as calculated by Spectronaut by mass spectrometry after affinity purification of any of the baits. Preys are displayed on the *y* axis, and baits are displayed on the *x* axis. **b**, Bar graphs displaying GO analysis of preys detected by mass spectrometry after affinity purification of the unexpected hits from the tethering assay. Significantly enriched preys for GO analysis are defined as having *q* < 0.05 and log_2_ ratio IP/FLAG > 1 (multiple hypothesis corrected, determined by Spectronaut algorithm). Splicing-associated GO terms are highlighted in red. Gaps in the *y* axis are used to visualize the most highly enriched splicing-associated GO term when one was not present in the top four. Unadjusted *P* value of enrichment was calculated using Metascape^56^ based on the accumulative hypergeometric distribution. **c**,**d**, Stacked bar graphs displaying the overall count of preys significantly detected in follow-up experiments over IgG controls (fold change > 0.5, unadjusted *P* < 0.00000001 as determined by the Spectromine algorithm), separated by interaction type (Methods). The count of preys that are annotated as RBPs (**c**) and contain RNA splicing GO terms (**d**) are displayed.

**Extended Data Fig. 5:**
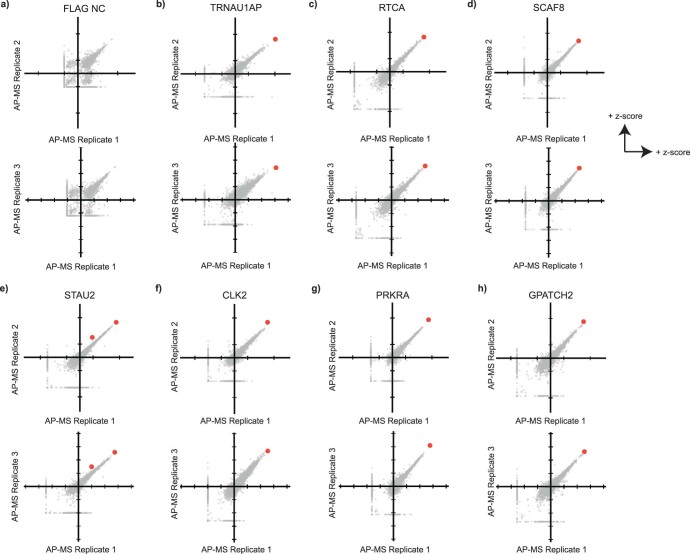
Quality control of AP-MS. **a-h** Scatter plots showing concordance between AP-MS replicates. Each point represents a detected protein and its z-score in two replicates per plot. Red points represent the detection of the bait protein among the preys. Multiple red points indicate multiple major isoforms detected with average Z-score>1.

Splicing occurs through assembly and action of complexes consisting of multiple proteins and RNAs, including core spliceosomal components and non-essential splicing factors. To examine if splicing-associated proteins interact with our candidates, we performed affinity purification–mass spectrometry (AP–MS) of V5-tagged TRNAU1AP, RTCA, SCAF8 and STAU2 expressed in HEK293T cells (Fig. 4 and Supplementary Table 17). We performed AP–MS in the absence of ribonuclease, allowing the detection of both proteins that interact directly with our candidates as well as proteins that our candidates associate with through nearby binding on RNA substrates. We aimed to include these RNA-mediated associations, because mutual binding to the snRNAs of the spliceosome or nearby splice sites on mRNA can indicate interactions during splicing. Replicates were highly correlated, and each bait protein was present among the top preys in corresponding samples (Extended Data Fig. 5). We also performed AP–MS with a known splicing-associated protein (CLK2), a tag-only control (FLAG-V5) and two RBPs from the screens that did not emerge as hits (PRKRA and GPATCH2).

We examined the enrichment of splicing-associated proteins (annotated with GO:0008380 RNA splicing, GO:0005681 Spliceosomal Complex or any of their child terms) in each of the AP–MS samples that were significantly enriched (*z*-score > 2) in at least one of the AP–MS samples (Fig. 4a). Setting aside the tag-only control, the baits separated into two clusters, one with high enrichment of splicing-associated proteins among the preys and the other with low enrichment. The low-enrichment cluster consists of the two non-activating controls and STAU2. Nevertheless, STAU2 is still enriched for interactions with a subset of splicing-associated proteins over the non-targeting controls, potentially due to it performing a limited, auxiliary role in splicing. The high-enrichment cluster consists of the known splicing-associated protein CLK2 as well as TRNAU1AP, SCAF8 and RTCA, candidates that also displayed widespread direct modulation of AS of endogenous targets. Overall, the increased enrichment of splicing-associated proteins in the TRNAU1AP, SCAF8 and RTCA AP–MS samples provides supporting evidence for them performing widespread splicing regulation.

We also performed GO enrichment on the significantly enriched preys as detected by Spectronaut (*q* < 0.05 and log_2_ ratio IP/FLAG > 1) with each of the candidates as bait (Fig. 4b). The splicing-associated GO term ‘regulation of mRNA splicing, via spliceosome’ was among the most highly enriched in the significantly enriched preys pulled down by TRNAU1AP and SCAF8. No splicing-associated GO terms were enriched among the significantly enriched preys pulled down by RTCA. The splicing-associated GO term ‘regulation of mRNA splicing, via spliceosome’ was enriched in the preys pulled down by STAU2 but was not among the top terms. Following the initial evidence of splicing-associated protein enrichment after TRNAU1AP, SCAF8 and RTCA pulldown, we matched these experiments with ribonuclease-positive conditions as well as matching IgG controls in ±ribonuclease conditions to distinguish between direct protein–protein interactions and RNA-mediated interactions (Fig. 4c,d)^51^. We applied a strict *P* value cutoff of 0.00000001 to visualize the most specific RBPs and splicing-associated proteins pulled down by each bait. The unfiltered output from follow-up experiments can be found in Supplementary Table 18. Overall, we used AP–MS to indicate that splicing-associated proteins are enriched after pulldown of TRNAU1AP, SCAF8 and RTCA and to identify the specific modes by which these proteins interact with RBPs and splicing-associated proteins.

### AS modulation by TRNAU1AP

Owing to strong evidence across the eCLIP, knockdown RNA-seq and AP–MS data indicating the activity of TRNAU1AP as a splicing factor, we examined the protein in further detail. We first investigated the finding that most genes with TRNAU1AP knockdown-sensitive skipped exon events did not contain reproducible enriched binding windows from the eCLIP data. We considered the hypothesis that some of this effect could be explained by TRNAU1AP indirectly regulating splicing events through modulating the splicing of other splicing factors. This multi-layered control of splicing has been shown in the recently characterized splicing factor DAP3 (ref. ^52^) as well as in the SR family of splicing factors^20^. To investigate this, we examined the top differentially expressed and differentially spliced genes with RNA splicing GO terms (splicing-associated genes) after TRNAU1AP knockdown.

**Fig. 5:**
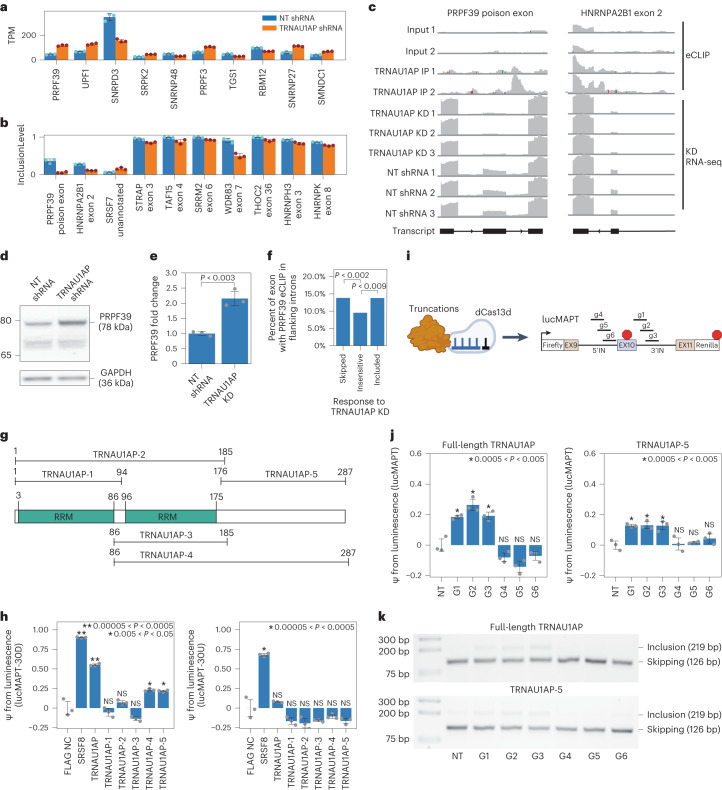
TRNAU1AP participates in splicing co-regulatory networks and activates exon inclusion through a C-terminal effector domain. **a**, Bar graph showing relative expression level of the top 10 differentially expressed splicing-associated genes as sorted by DeSeq2-determined adjusted *P* value after TRNAU1AP knockdown (mean ± s.d., *n* = 3 replicate transductions). **b**, Bar graph showing relative exon inclusion level of the top 10 differentially spliced skipped exon events in splicing-associated genes as sorted by rMATS-determined adjusted *P* value after TRNAU1AP knockdown (mean ± s.d., *n* = 3 replicate transductions). **c**, IGV browser tracks showing coverage of TRNAU1AP eCLIP signal relative to size-matched input and TRNAU1AP knockdown RNA-seq signal relative to non-targeting shRNA at a poison exon in PRPF39 and exon 2 of HNRNPA2B1. **d**, Representative western blot showing increased PRPF39 expression in HEK293T cells after TRNAU1AP knockdown. GAPDH is the loading control. **e**, Bar graph showing fold change of PRPF39 expression as quantified by western blot after TRNAU1AP knockdown (mean ± s.d., *n* = 3 replicate transfections). *P* = 0.0024 by two-tailed independent two-sample *t*-test. **f**, Bar plot displaying percentage of exons containing PRPF39 reproducible enriched eCLIP windows in flanking introns from ENCODE HepG2 data, separated by exon sensitivity to TRNAU1AP knockdown in HEK293T cells. *P* values are calculated using the two-sided chi-squared test. *P* = 0.0011 for PRPF39 binding to exons skipped after TRNAU1AP knockdown and 0.0088 for exons included after TRNAU1AP knockdown. **g**, Domain structure of TRNAU1AP with truncations used for effector domain identification. **h**, Bar graphs displaying reporter readout from both lucMAPT-30U and lucMAPT-30U co-transfected with MCP-fused truncations (mean ± s.d., *n* = 3 replicate transfections). *P* value was calculated by one-tailed independent two-sample *t*-test. NS, not significant (*P* > 0.05). **i**, Schematic of truncation–dCas13d fusions used as for MS2-free tests. Schematic of MS2-free lucMAPT reporter used and associated guide RNAs. **j**,**k**, Reporter readouts from co-transfection of the MS2-free lucMAPT reporter, either full-length TRNAU1AP–dCas13d fusion or truncated TRNAU1AP-5–dCas13d fusion, and each guide RNA annotated in **i**. **j**, Bar graph showing PSI calculated from luminescence (mean ± s.d., *n* = 3 replicate transfections). *P* value was calculated by one-tailed independent two-sample *t*-test. NS, not significant (*P* > 0.05). **k**, Splicing gels displaying lucMAPT AS. bp, base pairs; KD, knockdown.

**Extended Data Fig. 6:**
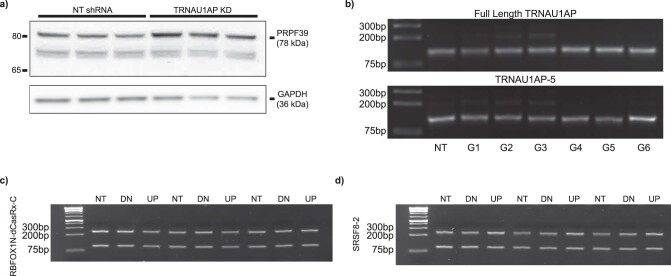
Full western blots and splicing gels for TRNAU1AP follow-up experiments and modulation of endogenous HNRNPD Exon 7. **a** Western blot replicates used for quantification showing increased PRPF39 expression in HEK293T cells following TRNAU1AP knockdown. GAPDH is the loading control. n = 3 independent transductions. **b** Additional replicate displaying lucMAPT alternative splicing from co-transfection of the MS2-free lucMAPT reporter, either full-length TRNAU1AP-dCas13d fusion or truncated TRNAU1AP-5-dCas13d fusion, and each reporter targeting guide RNA annotated in Fig. 5i. n = 2 independent transfections **c-d** Agarose gels of amplified cDNA collected from HEK293T cells co-transfected with artificial splicing factors (RBFOX1-dCasRx-C, SRSF8-2) and gRNA arrays (NT = non-targeting gRNA, DN = downstream 3-gRNA array, UP = upstream 3-gRNA array). n = 3 independent transfections. Source data

The top differentially expressed splicing-associated gene was *PRPF39* (Fig. 5a), and the top two differentially spliced splicing-associated genes were *PRPF39* (at an unannotated poison exon) and *HNRNPA2B1* (at exon 2, responsible for isoform switching between HNRNPA2 and HNRNPB1) (Fig. 5b). In TRNAU1AP knockdown, presence of the *PRPF39* poison exon is virtually eliminated, and *PRPF39* TPM increases from 46.06 ± 3.62 to 117.34 ± 5.06 (mean ± s.d.). TRNAU1AP binds in the intron downstream on this poison exon (Fig. 5c, left). We performed western blots to validate that the increase in PRPF39 expression after TRNAU1AP knockdown is reflected at the protein level and detected a two-fold increase in HEK293T cells (Fig. 5d,e and Extended Data Fig. 6a). Due to the extent of poison exon elimination in the knockdown condition, TRNAU1AP appears to be the primary driver of poison exon-mediated expression control of PRPF39 in HEK293T cells. As an initial investigation to test the hypothesis of PRPF39 acting as a direct effector for certain TRNAU1AP knockdown-sensitive AS events, we analyzed PRPF39 eCLIP signal in HepG2 cells generated by the ENCODE consortium^45^. We found that PRPF39 reproducible enriched binding windows are prevalent in a significantly higher percentage of introns flanking TRNAU1AP-sensitive exons than TRNAU1AP-insensitive exons, supporting the hypothesis (Fig. 5f). We also examined another TRNAU1AP-sensitive splicing factor exon, *HNRNPA2B1* exon 2, which also contains TRNAU1AP binding sites in the downstream intron and is virtually eliminated in TRNAU1AP knockdown (Fig. 5b,c, right). This implicates TRNAU1AP as the primary driver of isoform switching of *HNRNPA2B1* in HEK293T cells. Here, we showed that TRNAU1AP binds to the downstream intron of, and drives the inclusion of, exons in *PRPF39* and *HNRNPA2B1*, which likely drives further widespread splicing changes.

To identify the effector domain bestowing TRNAU1AP’s ability to drive exon inclusion, we then performed a series of truncation experiments. We cloned truncations (Fig. 5g) into MCP fusions using the same backbone as the RBP library in the initial tethering screen. We co-transfected MCP-fused TRNAU1AP truncations with both splicing reporters, attempting to identify the region of the protein sufficient to drive the downstream-only effect captured in the screen (Fig. 5h). The C-terminal domain captured in truncations TRNUA1AP-4 and TRNUA1AP-5 appears to be responsible for most, but not all, of the exon inclusion driving activity of the full-length protein. This allowed us to build a domain model that matches the standard simplified model of an RBP, consisting of independent and separate effector and binding domains—in this case, an RNA-binding RRM-containing domain at the N-terminus and an exon inclusion activating effector domain at the C-terminus.

To ensure that the exon-including capacity of TRNAU1AP and its C-terminal effector domain is not dependent on the MS2–MCP interaction, we cloned CRISPR artificial splicing factors by fusing TRNAU1AP-5 and full-length TRNAU1AP to catalytically dead Cas13d. We co-transfected these artificial splicing factors with a version of the lucMAPT splicing reporter lacking MS2 stem loops, along with individual gRNA plasmids targeting the introns upstream and downstream of the alternatively spliced exons (Fig. 5i). Both full-length TRNAU1AP and TRNAU1AP-5 significantly drove exon inclusion as measured by the tethering-free reporter when co-transfected with gRNAs targeting downstream of the alternatively spliced exon but not with those targeting upstream (Fig. 5j,k and Extended Data Fig. 6b). These results are consistent with the downstream-only result from the tethering assays and show that the ability of TRNAU1AP and its C-terminal effector domain to induce exon inclusion is independent of the MS2–MCP interaction. In summary, we show that TRNAU1AP participates in splicing co-regulatory networks and drives exon inclusion through its C-terminal effector domain.

### Employing identified domains in artificial splicing factors

**Fig. 6:**
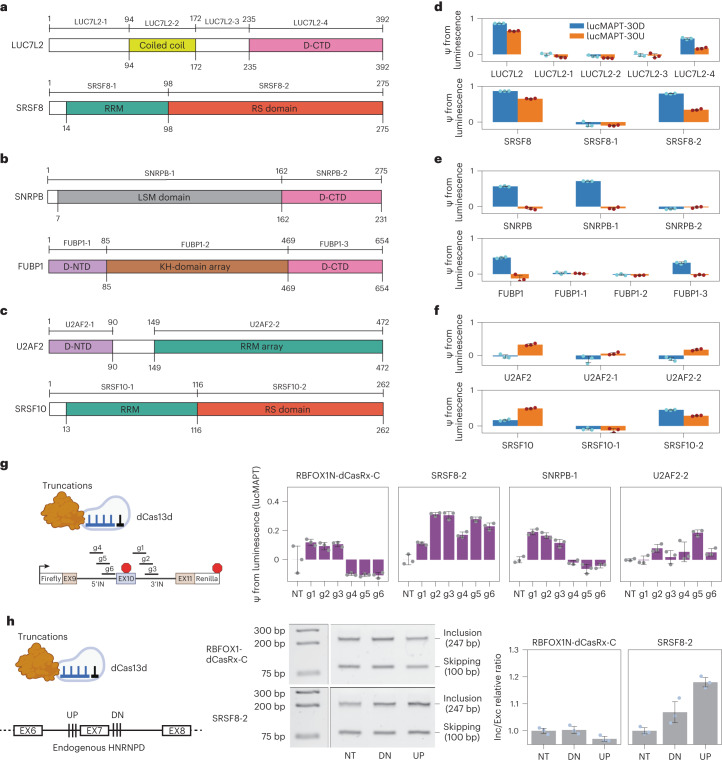
Truncation of the top RBP hits identify splice-enhancing domains that can be repurposed for artificial splicing factors. **a**–**c**, Domain structures of top hits used for truncation experiments; D-NTD and D-CTD represent N-terminal and C-terminal domains, respectively, containing MobiDB-lite consensus disorder prediction. All tested truncations are shown. Hits are separated into their position dependence from the initial screen: position-independent hits (**a**), hits that primarily activated the lucMAPT-30D reporter (**b**) and hits that primarily activated the lucMAPT-30U reporter (**c**). **d**–**f**, Bar graphs displaying reporter readout from both lucMAPT-30U and lucMAPT-30U of the full-length proteins next to their associated truncations (mean ± s.d., *n* = 3 replicate transfections). Graphs are separated by position dependence of full-length protein from the initial screen: position-independent hits (**d**), hits that primarily activated the lucMAPT-30D reporter (**e**) and hits that primarily activated the lucMAPT-30U reporter (**f**). **g**, Left, top, schematic of truncation–dCas13d fusion used as artificial splicing factors. Left, bottom, schematic of MS2-free lucMAPT reporter used for reporter-based assessment of artificial splicing factors. Right, bar graphs displaying reporter output from MS2-free lucMAPT reporter after co-transfection of reporter with truncation–dCas13d fusion and gRNA-containing plasmid (mean ± s.d., *n* = 3 replicate transfections). **h**, Left, top, schematic of truncation–dCas13d fusion used as artificial splicing factor. Left, bottom, schematic of HNRNPD exon 7 used for endogenous splicing modulation, with the position of the two sets of three gRNAs that are co-transfected with the artificial splicing factors as gRNA arrays. Middle, agarose gel showing splicing of HNRNPD exon 7 of a sample replicate for both artificial splicing factors in co-transfection with both gRNA arrays and a non-targeting gRNA (NT). Right, bar graphs displaying quantification of inclusion/exclusion ratio normalized to the non-targeting gRNA (NT) from gels in Extended Data Fig. 6c,d (mean ± s.d., *n* = 3 replicate transfections). bp, base pairs.

Motivated by our results articulating that TRNAU1AP or its domain can be useful in artificial splicing factors, we returned to the original list of top RBPs that altered splicing of our reporter construct and tested various protein truncations of these with the aim of determining minimal splice-activating domains to repurpose for artificial splicing factors. LUC7L2 and SRSF8 were selected as strong hits that activated splicing both upstream and downstream of the alternative exon (Fig. 6a). SNRPB and FUBP1 were selected as strong hits that activated lucMAPT-30D only (Fig. 6b). U2AF2 and SRSF10 were selected as strong hits that primarily activated exon inclusion when tethered upstream (Fig. 6c). We designed and cloned truncations based on domain structure, assuming modularity of RBPs where effector and binding domains are separate and independent.

Selected truncations were fused to the MS2 coat protein using the same backbone and conditions as the RBP–MCP library (Fig. 6d–f). LUC7L2-4 recapitulated some of the activity of its full-length counterpart, however at substantially lower strength, implying important contributions from the other domains. SRSF8-2, the RS domain of the protein, captured much of the activity of SRSF8. FUBP1-3 captured much of the activity of full-length FUBP1, at a markedly reduced size. SNRPB-1 captured all the activity of SNRPB. Interestingly, SRSF10-2, the RS domain of SRSF10, displayed a different modulation pattern than the full-length protein, where a stronger effect was seen when tethered downstream of the alternatively spliced exon, more in line with all other tested SRSF proteins. U2AF2-2 was the most successful truncation of the proteins that activated only lucMAPT-30U.

We constructed CRISPR-based artificial splicing factors by fusing the truncations that most successfully activated the tethering reporter to catalytically dead Cas13d. These were tested with an MS2-free luciferase splicing reporter and compared with the recently reported RBFOX1N-dCasRx-C artificial splicing factor^19^ (Fig. 6g). As expected, RBFOX1N-dCasRx-C activated the reporter only when targeting sites downstream of the alternatively spliced exon, with a maximal ψ of 11.87% with g1. The SRSF8-2-based artificial splicing factor activated the reporter at all positions, with a maximal ψ of 31.34% with g2. The SNRPB-1-based artificial splicing factor activated the reporter only when targeting downstream of the alternatively spliced exon, as for RBFOX1N-dCasRx-C, but with a greater maximal ψ of 19.15% with g1. The U2AF2-2-based artificial splicing factor did not show activation only with upstream gRNAs as expected, although activation was maximized with upstream guide g5 at 18.60%. Altogether, the SNRPB-1 artificial splicing factor directly outperformed RBFOX1N-dCasRx-C; the SRSF8-2 artificial splicing factor provided a stronger tool with reduced position dependence; and the U2AF2 artificial splicing factor introduced a tool with upstream position association.

Activation of endogenous exon inclusion has remained challenging for the field, as the current solutions with antisense oligonucleotides (ASOs) are to block splicing repressor sites, which is not generalizable to exons that lack these. We employed a CRISPR artificial splicing factor based on our strongest activation domain, SRSF8-2, against an endogenous exon. We targeted exon 7 of *HNRNPD* in HEK293T cells, selected for its high expression for facile readout and endogenous inclusion rate of roughly 50% for perturbation detection. We compared our SRSF8-2 artificial splicing factor to the previous RBFOX1N-dCasRx-C artificial splicing factor by co-transfecting each with plasmids containing arrays of three gRNA sequences separated by repeats that are processed by Cas13d into independent guides. RBFOX1-dCasRx-C was not able to activate endogenous *HNRNPD* exon 7 inclusion with either of the gRNA arrays, whereas SRSF8-2 was able to with both arrays, especially the upstream array (Fig. 6h and Extended Data Fig. 6c,d). Exon 7 of *HNRNPD* appears to be most sensitive to inclusion, driving perturbation with effector domains guided to the upstream 3′ splice site, which is incompatible with the downstream-only effect of RBFOX1-dCasRx-C but can be driven by SRSF8-2, exemplifying the importance of its generalizability. Furthermore, the stronger SRSF8-2 appeared to cross an activation threshold when guided to the downstream 5′ splice site, whereas the weaker RBFOX1-dCasRx-C did not. In summary, our tethering assay and reporter system also allowed us to identify small and potent effector domains that we used to improve synthetic splicing modulatory proteins.

## Discussion

We developed tethering assays and used these to assess the ability of 718 RBPs to induce exon inclusion after recruitment nearby an alternatively spliced cassette exon. Of the 718 RBPs evaluated, 58 reliably enhanced inclusion. Forty-seven of these 58 were annotated with splicing-associated GO terms, and 11 of these were previously unknown as performing any role in AS. We further applied our assays for technology development by using them to rapidly test exon inclusion activation domains identified from the top candidates for use in engineered splicing factors. By fusing these identified domains to catalytically dead Cas13d, we built CRISPR-based artificial splicing factors that are smaller, more potent and less restricted than current technologies. Our tethering assays served as fast, scalable and reliable platforms for both applications.

We employed eCLIP, AP–MS and shRNA knockdown followed by RNA-seq to endogenous TRNAU1AP, SCAF8, RTCA and STAU2, which, excitingly, provided evidence for regulation of splicing outcomes. We further implicated TRNAU1AP as a multi-layered regulator of splicing that also acts in splicing regulatory networks by modulating the splicing of other splicing factors. We performed AP–MS in ribonuclease-free conditions and detected splicing-associated proteins after pulldown of TRNAU1AP, RTCA and SCAF8, further supporting their role in splicing. Findings here are limited by the sensitivity and specificity of the assays chosen as well as potential tissue specificity of effects on splicing of the chosen proteins. Future work should investigate the role of these proteins on splice site selection in orthogonal models and employ further validation approaches, such as minigene assays of specific splicing events and co-IP western blots, to validate interaction partners.

Furthermore, the functional consequences of splicing modulation by TRNAU1AP, SCAF8, RTCA and STAU2 in health and disease remain to be investigated. The splicing regulatory network formed by TRNAU1AP and PRPF39 deserves further investigation. TRNAU1AP and PRPF39 were recently identified as a co-dependency module that is selectively essential in cells carrying mutational signatures of DNA mismatch repair^53^. The interaction of TRNAU1AP regulating PRPF39 expression through poison exon inclusion described here provides a mechanistic hypothesis for this finding. Furthermore, both genes are prognostic markers in a variety of cancer types^54^. As our scope is limited to the introduction and initial characterization of these proteins in splicing regulation, we are excited for future investigations.

Our SNRPB-1 artificial splicing factor maintained the downstream targeting specificity of the prior RBFOX1N-dCasRx-C artificial splicing factor but with higher potency and a reduced size. We also identified exon activation domains with different specificity requirements. Our U2AF2-2 artificial splicing factor has maximum potency when targeted upstream of an AS exon, whereas our SRSF8-2 artificial splicing factor is the strongest thus far and maintains potency with proximity to the AS exon independent of orientation. This orientation independence proved important in our targeting of endogenous *HNRNPD* exon 7, where SRSF8-2 successfully activated exon inclusion and RBFOX1N-dCasRx-C did not.

A limitation of our assays is the potential of false negatives, and RBPs testing negative could still play a role enhancing exon inclusion in different contexts. Our work with lucMBNL1 exemplifies this by demonstrating a sequence context around an AS exon that responds only to a small subset of RBPs that induced exon inclusion in lucMAPT. Future studies that employ tethering approaches in a variety of minigene contexts could identify additional hits with different RNA sequence requirements. Loss of function due to the C-terminal MCP fusion might also explain false negatives in our screens. Nevertheless, these assays have provided the first of possibly many comprehensive investigations of proximity-dependent direct activators of exon inclusion. As the reporters were, to a small extent, sensitive to NMD, caution should be raised when using them in applications across different NMD environments or in applications that may detect changes in the processing of mature reporter mRNA. However, there is potential for NMD sensitivity to be engineered away in future versions of the reporter by relying on alternative exon-induced frameshift to halt translation in the final constitutive exon as opposed to introducing a stop codon in the alternative exon.

We anticipate utility in future studies from our methodology in large-scale discovery of RBPs that enhance exon inclusion by proximity, from our introduction and molecular characterization of previously uncharacterized AS proteins and from our development of small and potent molecular parts for engineered splicing modulation. Future studies could be used to examine the ~2,000 predicted human RBPs not included in our assays. Our engineered splicing domains can be used in future work for delivery through adeno-associated virus (AAV) with their reduced size over current technologies in models incompatible with transfection, and the increased potency can lower dose requirements and expand applicability of the technology. These minimal and potent splicing domains can also be recruited to RNA targets through other means than dCas13d, such as through PUF proteins^18^ or CRISPR–Cas-inspired RNA targeting systems (CIRTS)^55^. Altogether, we are optimistic that future approaches will leverage the principles presented here to further explore the landscape of splicing regulation.

## Methods

### Generation of expression plasmids for MCP and dCas13d-fused RBPs and RBP truncations

Most ORF clones were obtained in pENTR vectors from the CCSB human ORFeome collection^58^ (Dana-Farber Cancer Institute) or the DNASU Plasmid Repository (Arizona State University). For truncations, domain structures were determined using InterProScan^59^ on the amino acid sequence of the full-length protein and informed truncation design. Truncations and ORFs that were ordered in standard expression vectors were amplified by PCR (Phusion polymerase, New England Biolabs (NEB)) with oligonucleotide primers containing attB recombination sites and recombined into pDONR221 using BP clonase II (Thermo Fisher Scientific). ORFs were then recombined into one of two custom pEF DEST51 destination vectors (Thermo Fisher Scientific). For MCP fusions, the destination vector is engineered to direct expression of the ORFs as fusion proteins with a V5 epitope tag and MCP appended C-terminally and under the control of the EF1-alpha promoter to create ORF–V5–MCP constructs. For dCas13d fusions, the MCP is simply replaced with dCas13d for the generation of ORF–V5–dCas13d constructs. Supplementary Table 19 contains sequences of both destination vectors. The identity of all cDNA clones was verified by Sanger sequencing. Plasmid libraries are available on Addgene (155390–156159). Supplementary Table 1 lists all ORFs and relevant information.

### Cell lines

Lenti-X HEK293T cells were purchased from Takara Bio and were not further authenticated. Cells were routinely tested for mycoplasma contamination with a MycoAlert mycoplasma test kit (Lonza) and were found negative for mycoplasma.

### Generation of constructs

#### lucMAPT reporter

Reporter was first constructed through a three-fragment Gibson Assembly using a homebrew enzyme mix (OpenWetWare). Fragments were generated by performing PCR on sub-fragments to generate complementary overhangs, followed by annealing, amplification and agarose gel extraction. The first fragment consists of Firefly luciferase, MAPT exon 9 and the 5′-most 500 base pairs of MAPT intron 9. The second fragment consists of the 3′-most 500 base pairs of MAPT intron 9, modified MAPT exon 10 and the 5′-most 500 base pairs of MAPT intron 10. The third fragment consists of the 3′-most 500 base pairs of MAPT intron 10, MAPT exon 11 and Renilla luciferase. Luciferase ORFs were cloned from plasmids used in our laboratory’s previous work^16^. MAPT exons were ordered as synthetic oligonucleotides. MAPT intronic sequences were amplified from genomic DNA isolated from Lenti-X HEK293T cells. All PCR was performed using KAPA HiFi HotStart ReadyMix (Roche, 7958935001). The assembly strategy is summarized in Extended Data Fig. 1a.

#### lucMAPT–MS2 reporters

MAPT exon 10 and the flanking 100 intronic base pairs in either direction from the splice sites were removed from the construct and replaced with a cloning site containing BamHI and EcoRI cut sites through PCR, followed by two-fragment Gibson Assembly to generate a customizable backbone. Inserts containing MAPT exon 10, the flanking 100 base pairs and the MS2 stem-loop sequence in the desired position were cloned into this backbone through one-fragment Gibson Assembly into pcDNA3.1 (−) Mammalian Expression Vector (Thermo Fisher Scientific, V79520) to construct lucMAPT–MS2 reporters. Inserts containing other AS exons and flanking sequences were used to generate other reporters used. Sequences of reporters can be found in Supplementary Table 19.

### Luciferase reporter screens

#### Reverse transfection

Ninety-six-well Solid Black Flat Bottom Polystyrene TC-treated Microplates (Corning, 3916) were coated with 75 μl of poly-d-lysine hydrobromide (Sigma-Aldrich, P6407-5MG), dissolved in water at 1 g L^−1^ and further diluted 1:5 in 1× DPBS (Corning, 21-031-CV) overnight in a tissue culture incubator. Plates were rinsed two times with 1× DPBS and dried. A 1:1 mix of lucMAPT–MS2 reporter and an ORF–V5–MCP construct with a total of 100 ng of DNA were added to a mixture of Lipofectamine 3000 and P3000 reagents (Thermo Fisher Scientific, L3000001), diluted in Opti-MEM Reduced Serum Media (Gibco, 31985062) and incubated for 15 min. The mixture of DNA and transfection reagent was transferred to the PDL-coated 96-well plate. Then, 75 μl of Lenti-X HEK293T cells was plated at a concentration of 266,666 cells per milliliter. Transfection was incubated for 48 h in a standard tissue culture incubator.

#### Dual-luciferase readout

Luminescence was generated using the Dual-Glo Luciferase Assay System (Promega, E2980). Cells were removed from the incubator to cool to room temperature for 30 min. Then, 75 μl of Dual-Glo Luciferase Reagent was added directly to cells and thoroughly mixed using a Microplate Genie Plate Shaker (Scientific Industries). The reaction was briefly centrifuged and allowed to incubate at room temperature for 10 min. Luminescence was measured using a Spark Multimode Microplate Reader (Tecan) with a 500-ms signal interaction time at room temperature. The same process was repeated for Renilla luciferase luminescence using the Dual-Glo Stop & Glo Reagent.

#### Statistical analysis

Relative ψ values were calculated as described in Fig. 1b using the pandas library in Python version 3.10.11 (ref. ^60^). All plots generated from Python were generated using JupyerLab 4.04. Significance between candidate and negative control conditions was assessed by calculating *P* value through a one-tailed independent *t-*test using the ttest_ind function in scipy^61^.

### RNA-level validation of luciferase screens

Transfection was performed as described for the luciferase reporter screens, using standard 96-well tissue culture plates (Costar, 3596). RNA was isolated from cells using the Direct-zol RNA Miniprep Kit (Zymo Research, R2052). cDNA was generated using the ProtoScript II First Strand cDNA Synthesis Kit (Promega, E6560L). cDNA was amplified using GoTaq Green Master Mix (Promega, M7122), and primers were designed for an amplicon stretching from MAPT exon 9 to the Renilla luciferase ORF. Amplicons were run through a 3% SeaKem Agarose Gel (Lonza, 5004) at 100 V for 25 min.

#### Statistical analysis

Relative band intensity was calculated using the Gel Analyzer feature in ImageJ version 1.53k software^62^. Significance between candidate and negative control conditions was assessed by calculating *P* value through a one-tailed independent *t-*test using the ttest_ind function in scipy^61^.

### GO analysis

Metascape version 3.5 was used for GO analysis^56^. Custom enrichment analysis for GO Biological Processes was performed using an appropriate set of background genes. biomaRt version 2.50.3 was used to identify genes matching specific GO terms from gene lists^63^. We used biomaRt to generate a list of splicing associated genes by selecting genes annotated with GO:0008380 RNA splicing, GO:0005681 Spliceosomal Complex or any of their child terms.

### Generation of samples overexpressing V5-tagged RBPs

HEK293T cells were plated in 10-cm plates at 10% confluency. Then, 28 ng of plasmid DNA encoding the V5-tagged RBPs was added to a mixture of Lipofectamine 3000 and P3000 reagents (Thermo Fisher Scientific, L3000001), diluted in Opti-MEM Reduced Serum Media (Gibco, 31985062) and incubated for 15 min. The mixture of DNA and transfection reagent was transferred to the plated cells. Cells were collected 48 h later and washed with 10 ml of DPBS. Samples to be used for eCLIP were UV cross-linked (400 mJ cm^−2^, 254 nm). Cells were resuspended in 1 ml of DPBS. Samples were centrifuged at 4 °C and 18,000*g* for 1 min. Supernatant was removed, and cells were flash frozen in dry ice before storage at −80 °C until experimentation.

### eCLIP library preparation and sequencing

eCLIP was performed as per Yeo laboratory standard operating procedures^44^. Antibodies used are listed in Supplementary Table 20. For V5-tagged eCLIPs, overexpression samples were generated as described herein. Samples for endogenous eCLIP were generated using the same procedure without transfection. Two replicates were generated for each experiment. Pellets were lysed, and lysates were subjected to sonication and RNase I to fragment RNA. Ninety-eight percent of each lysate was immunoprecipitated using either V5 (Bethyl, A190-120A) or TRNAU1AP-specific (GeneTex, GTX121631) antibodies, and the remainder was stored for preparation of a SMInput library. Ten micrograms of antibody was used per sample. Pulled-down RNA fragments were dephosphorylated and 3′-end ligated to an RNA adaptor. Immunoprecipitates and SMInputs were run on an SDS-polyacrylamide gel and transferred to a nitrocellulose membrane. Membrane regions from the RBP size to that size plus 75 kDa were excised, and RNA was released with proteinase K. SMInput samples were then dephosphorylated and 3′-end ligated to an RNA adaptor. All samples were reverse transcribed with SuperScript III Reverse Transcriptase (Life Technologies). cDNAs were ligated to a DNA adaptor at the 5′ end. cDNA was quantified by qPCR and amplified to 100–500 fmol of library using Q5 PCR Master Mix (NEB). Sequencing was performed using the NovaSeq 3000 platform, with a targeted number of single-ended reads of 40 million per sample.

### Computational analysis of eCLIP data

Computational analysis of eCLIP data was performed using the default settings of Skipper resources available on GitHub (https://github.com/YeoLab/skipper). Reads were mapped to human genome assembly GRCh38 (ref. ^64^). For V5-tagged eCLIPs, reproducible enriched windows were first found after transfection and eCLIP of a V5-FLAG negative control plasmid and added to the blacklist file to reduce spurious enrichment from V5 binding to RNA.

### shRNA lentiviral production, transduction and sequencing

To generate lentiviral particles for RBP knockdown, we seeded 500,000 HEK293T cells per well in six-well plates. After 24 h, cells in each well were transfected with 500 ng of sequence-verified shRNA plasmid (pLKO.1; Supplementary Table 21) and packaging plasmids (50 ng of pMD2.G: Addgene, 12259; 500 ng of psPAX2: Addgene, 12260—both gifts from Didier Trono, École polytechnique fédérale de Lausanne) using Lipofectamine 3000 (Thermo Fisher Scientific). Transfection media was replaced with 2.5 ml of fresh media after 6 h. Virus-containing medium was collected 48 h later, replaced with 2.5 ml of fresh media and collected again a further 24 h later. Virus-containing media were pooled and stored at −80 °C until transduction.

For lentiviral transduction, 500,000 HEK293T cells were seeded per well in each well of a six-well tissue culture plate. After 24 h, media were replaced with 2 ml of virus-containing media supplemented with 16 µg of polybrene. We replaced the virus-containing media with fresh media 24 h later. Twenty-four hours after this, media were replaced with fresh media containing 3 µg ml^−1^ puromycin. Cells were either given fresh puromycin-containing media or passaged every 48 h and expanded to 10-cm plates. Cells were pelleted and flash frozen once all replicates for a given construct had reached 70% confluency or higher.

Total mRNA was extracted from samples using the Direct-zol RNA Miniprep Kit (Zymo Research). RNA quality was verified using TapeStation 3000 (Agilent Technologies). Library preparation was performed using the Stranded mRNA Prep Ligation Kit (Illumina). Sequencing was performed using the NovaSeq 3000 platform, with a targeted number of paired ended reads of 60 million per sample. Read counts and uniquely mapped reads were verified after STAR version 2.6.7a alignment.

### Differential expression analysis

Differentially expressed genes were detected from RNA-seq data using DeSeq2 (ref. ^65^). We only considered genes expressed with TPM > 10 in the control sample.

### Differential splicing analysis

Differential AS events were detected using rMATS 4.0.2 (ref. ^66^). Splicing events were identified as significantly differentially spliced if the absolute value of inclusion-level difference was detected as greater than 5% and with a false discovery rate (FDR) of less than 5%. We only considered differential splicing events with a sum of ≥150 reads across all conditions.

### Integrated analysis of eCLIP and shRNA knockdown followed by RNA-seq data

The fraction of knockdown-sensitive or knockdown-insensitive genes containing binding sites from eCLIP was calculated using the number of genes expressed with TPM ≥ 10 from the eCLIP size-matched input as the denominator.

Binding position relative to knockdown-sensitive exons is visualized as the midpoint of the significantly enriched window. For events where multiple significantly enriched windows were present in a single feature, the midpoint of the median window is displayed.

### Western blots

Cells were lysed in lysis buffer (see eCLIP protocol) on ice for 15 min and sonicated for 5 min. Lysates were centrifuged at 15,000*g* for 10 min at 4 °C to pellet debris and transferred to a clean tube. Total protein concentration was quantified using the Pierce BCA Protein Assay Kit (Thermo Fisher Scientific, 23225). For gel electrophoresis, 20 μg was loaded per well onto 4–12% Bis-Tris gels and subsequently transferred to PVDF membranes. Membranes were blocked in 5% milk in TBST solution for 60 min at room temperature. Primary antibodies for UPF1 (Cell Signaling Technology, D15G6, 1:1,000), PRPF39 (Invitrogen, PA5-21627, 1:1,000) and GAPDH (Millipore, MAB374, 1:10,000) were diluted in 5% milk in TBST and probed overnight at 4 °C. Secondary antibodies (goat anti-rabbit IgG, HRP-linked, Cell Signaling Technology, 7074, and 800CW, goat anti-mouse IgG, Licor, 926-32210) were diluted at 1:2,000 in 5% milk in TBST and probed for 120 min at room temperature.

### AP–MS

HEK293T cells overexpressing V5-tagged RBPs were generated as described herein. Cells were lysed and affinity purified using 10 µg per sample of a V5-specific antibody. In brief, the cell lysates with antibody were incubated with magnetic beads overnight in the cold room. Then, 5 µl of 10 mg ml^−1^ RNase A was added to ribonuclease-positive conditions at this step. Supernatants were removed, and beads were washed four times with NP-40 buffer, twice in Buffer 2 (50 mM Tris (pH 7.5), 150 mM NaCl, 10 mM MgCl_2_, 0.05% NP-40 and 5% glycerol) and twice in Buffer 3 (50 mM Tris (pH 7.5), 150 mM NaCl, 10 mM MgCl_2_ and 5% glycerol). After the last wash, the wash buffer was aspirated completely, and the beads were resuspended in 80 μl of trypsin buffer (2 M urea, 50 mM Tris (pH 7.5), 5 μg ml^−1^ trypsin) to digest the bound proteins at 37 °C for 1 h with agitation. The beads were centrifuged at 100*g* for 30 s, and the partially digested proteins (the supernatant) were collected. The beads were then washed twice with 60 μl of urea buffer (2 M urea, 50 mM Tris (pH 7.5)). The supernatant of both washes was collected and combined with the partially digested proteins (final volume, 200 μl). After brief centrifugation, the combined partially digested proteins were cleared from residual beads. Then, 80 µl of these partially digested proteins was used; disulfide bonds were reduced with 5 mM dithiothreitol (DTT); and cysteines were subsequently alkylated with 10 mM iodoacetamide. Samples were further digested by adding 0.5 μg of sequencing-grade modified trypsin (Promega) at 25 °C. After 16 h of digestion, samples were acidified with 1% formic acid (final concentration). Tryptic peptides were desalted on C18 StageTips according to ref. ^67^ and evaporated to dryness in a vacuum concentrator and reconstituted in 15 μl of 3% acetonitrile/2% formic acid for liquid chromatography with tandem mass spectrometry (LC–MS/MS).

LC–MS/MS analysis was performed on a Q Exactive HF. Five microliters of total peptides was analyzed on a Waters M-Class UPLC using a 25-cm Thermo Fisher Scientific EASY-Spray column (2 µm, 100 A, 75 µm × 25 cm) coupled to a benchtop Thermo Fisher Scientific Orbitrap Q Exactive HF mass spectrometer. Peptides were separated at a flow rate of 400 nl min^−1^ with a 100-min gradient, including sample loading and column equilibration times. Data were acquired in data-independent (DIA) mode for initial experiments and data-dependent (DDA) mode for follow-up experiments. DIA MS1 spectra were measured with a resolution of 120,000, an automatic gain control (AGC) target of 5 × 10^6^ and a mass range from 350 *m*/*z* to 1,650 *m*/*z*; 34 isolation windows of 38 *m*/*z* were measured at a resolution of 30,000, an AGC target of 3 × 10^6^, normalized collision energies of 22.5, 25 and 27.5 and a fixed first mass of 200 *m*/*z*. DDA MS1 spectra were measured with a resolution of 120,000, an AGC target of 3 × 10^6^ and a mass range from 300 *m*/*z* to 1,800 *m*/*z*; MS2 spectra were measured at a resolution of 15,000, an AGC target of 1 × 10^5^, a TopN of 12, an isolation window of 1.6 *m*/*z* and a mass range from 200 *m*/*z* to 2,000 *m*/*z*.

Proteomics raw data were analyzed by Spectronaut version 16.0 (ref. ^68^) (Biognosys) using a UniProt database (*Homo sapiens*, UP000005640), and MS/MS searches were performed under Biognosys factory settings. UniProt GO term annotations (downloaded on 14 January 2022) were used for the differential enrichment analysis conducted by the Spectronaut software. Spectromine version 4.2.230428.52329 was used to analyze proteomics data in follow-up experiments using the same UniProt databases and default parameters. Preys identified in both the RNase treatment and non-treatment IPs for a particular bait were called ‘direct interactors’, and preys identified in only RNase non-treatment were called ‘RNA-mediated interactors’.

### Modulation of splicing with dCas13d fusions

Transfection was performed as described for the luciferase reporter screens. The plasmid DNA transfected consisted of 10 ng of lucMAPT Reporter DNA, 45 ng of gRNA plasmid and 45 ng of dCas13d–RBP fusion. Dual-luciferase readout was collected as described for the luciferase reporter screens. gRNA sequences were designed using the cas13design tool^69,70^. Transfection for modulation of endogenous targets was performed in 24-well plates with 250 ng of gRNA plasmid DNA and 250 ng of dCas13d–RBP fusion.

### Reporting summary

Further information on research design is available in the Nature Portfolio Reporting Summary linked to this article.

## Online content

Any methods, additional references, Nature Portfolio reporting summaries, source data, extended data, supplementary information, acknowledgements, peer review information; details of author contributions and competing interests; and statements of data and code availability are available at 10.1038/s41587-023-02014-0.

## Supplementary information

Reporting Summary

Supplementary Tables 1–21 Supplementary Table 1. For each RBP ORF in the screens, this table lists the location within the library, the GenBank gene symbol, the length of the ORF in nucleotides, the NCBI accession number, the nucleotide sequence and the amino acid sequence. Supplementary Tables 2–5. For each RBP tested in rounds 1 and 2 of the screens, these tables list the GenBank gene symbol, the NCBI accession number, the calculated ψ from the reporter measurement for each of three replicates, the mean, the standard deviation, the one-tailed unadjusted independent two-sample *t*-test-calculated *P* value of ψ, the location of the ORF within the library and the splicing reporter used. When multiple isoforms were present in the screens, the isoform that resulted in the stronger activation isoform was kept. lucMAPT-30D round 1 screen (Supplementary Table 2), lucMAPT-30U round 1 screen (Supplementary Table 3), lucMAPT-30D round 2 screen (Supplementary Table 4), lucMAPT-30D round 2 screen (Supplementary Table 5). Supplementary Tables 6 and 7. For each RBP ORF that passed round two of the screens, these tables list the position on the splicing gels at which the RBP was tested (Extended Data Fig. 1g), the GenBank gene symbol, the location of the ORF within the library, the ratio of inclusion band intensity to the sum of inclusion and skipping band intensities for each of two replicates, the mean, standard deviation, the one-tailed unadjusted independent two-sample *t*-test-calculated *P* value of the inclusion:(inclusion+skipping) ratio, the Bonferroni-adjusted *P* value cutoff, the pass state of each state of the ORF and the splicing reporter used. lucMAPT-30D splicing gels (Supplementary Table 6), lucMAPT-30U splicing gels (Supplementary Table 7). Supplementary Tables 8 and 9. For each RBP tested in the cross-validation experiments, these tables list the GenBank gene symbol, the NCBI accession number, the calculated ψ from the reporter measurement for each of three replicates, the mean, standard deviation, the one-tailed unadjusted independent two-sample *t*-test-calculated *P* value of ψ, the location of the ORF within the library and the splicing reporter used. lucMAPT-30D cross-validation experiments (Supplementary Table 8), lucMAPT-30U cross-validation experiments (Supplementary Table 9). Supplementary Table 10. For each candidate that passed all rounds of screening, this table lists the GenBank gene symbol, the NCBI accession number, the location of the ORF within the library, the reporter(s) that the candidate activated and the COMPARTMENTS confidence score for nuclear localization. Supplementary Tables 11–16. For each RBP tested in the orthogonal reporter experiments, these tables list the GenBank gene symbol, the NCBI accession number, the calculated ψ from the reporter measurement for each of three replicates in the experiment, the mean, standard deviation and the one-tailed unadjusted independent two-sample *t*-test-calculated *P* value of ψ, the location of the ORF within the library and the splicing reporter used. lucMAPT-100D experiments (Supplementary Table 11), lucMAPT-100U experiments (Supplementary Table 12), lucMBNL1-30D experiments (Supplementary Table 13), lucMBNL1-30U experiments (Supplementary Table 14), lucMAP3K7-30D experiments (Supplementary Table 15), lucMAP3K7-100U experiments (Supplementary Table 16). Supplementary Table 17. Results from the AP–MS experiments for eight baits composed of four unexpected hits (TRNAU1AP, SCAF8, STAU2 and RTCA), one non-splicing control (PRKRA), one positive splicing factor control (CLK2) and one background condition for the tagged IP (FLAG) are displayed in this table. For each gene detected in the overall experiment, this table shows the GenBank gene symbol, the UniProt ID and the average *z*-score across three replicates for each of the baits used. Supplementary Table 18. For each prey detected (fold change > 0.5 over IgG control, *P* < 0.05 as output by Spectromine) in the AP–MS follow-up experiments, this table lists the associated bait protein, the presence or absence of RNAse in the condition where the prey was detected, the average log_2_ ratio of the IP condition to the IgG control (*n* = 3), the unadjusted and multiple hypothesis-corrected *P* values as output by Spectromine of the IP/IgG ratio and descriptors of each prey. Supplementary Table 19. This table lists the sequences of the plasmids, primers and gRNAs used in this study. Supplementary Table 20. This table lists the target, vendor, catalog number, host species, application, dilution and ENCODE ID of antibodies used in this study. Supplementary Table 21. This table lists the sources and target sequences for the lentiviral shRNA constructs used in this study. TRC, The RNAi Consortium.

## Source data

Source Data Extended Data Fig. 1 Unprocessed western blot from Extended Data Fig. 1c.

Source Data Extended Data Fig. 6 Unprocessed western blot from Extended Data Fig. 6a. Both scans are from the same membrane.

## Data Availability

RNA-seq and eCLIP-seq data of this study are available at the National Center for Biotechnology Informationʼs Gene Expression Omnibus (accession code GSE232599)^71^. Source data are provided with this paper.
