# Author Correction: Large-scale evaluation of the ability of RNA-binding proteins to activate exon inclusion

**DOI:** 10.1038/s41587-024-02178-3

**Published:** 2024-02-28

**Authors:** Jonathan C. Schmok, Manya Jain, Lena A. Street, Alex T. Tankka, Danielle Schafer, Hsuan-Lin Her, Sara Elmsaouri, Maya L. Gosztyla, Evan A. Boyle, Pratibha Jagannatha, En-Ching Luo, Ester J. Kwon, Marko Jovanovic, Gene W. Yeo

**Affiliations:** 1 Department of Cellular and Molecular Medicine, University of California San Diego, La Jolla, CA USA; 2 Sanford Stem Cell Institute Innovation Center and Stem Cell Program, University of California San Diego, La Jolla, CA USA; 3 Institute for Genomic Medicine, University of California San Diego, La Jolla, CA USA; 4 Department of Bioengineering, University of California San Diego, La Jolla, CA USA; 5 Department of Biological Sciences, Columbia University, New York, NY USA

**Keywords:** Alternative splicing, Transcriptomics, High-throughput screening, Molecular engineering

Correction to: *Nature Biotechnology* 10.1038/s41587-023-02014-0, published online 2 January 2024.

In the version of the article initially published, the data in Fig. 6g were misaligned with the *x*-axis labels, displayed in incorrect 180° orientation. The figure has now been amended in the HTML and PDF versions of the article.

