# Supplementary material for: Large-scale evaluation of the ability of RNA-binding proteins to activate exon inclusion
Source: Nat Biotechnol. 2024 Jan 2;42(9):1429–41. doi: 10.1038/s41587-023-02014-0 (PMC11389820; doi:10.1038/s41587-023-02014-0)
Supplement: Supplementary file 1 — Reporting Summary [file 41587_2023_2014_MOESM1_ESM.pdf]

nature portfolio

Corresponding author(s): Gene W. Yeo

Last updated by author(s): Sep 27, 2023

Reporting Summary

Nature Portfolio wishes to improve the reproducibility of the work that we publish. This form provides structure for consistency and transparency in reporting. For further information on Nature Portfolio policies, see our Editorial Policies and the Editorial Policy Checklist.

Statistics

For all statistical analyses, confirm that the following items are present in the figure legend, table legend, main text, or Methods section.

n/a	Confirmed	
☐	☒	The exact sample size (*n*) for each experimental group/condition, given as a discrete number and unit of measurement
☐	☒	A statement on whether measurements were taken from distinct samples or whether the same sample was measured repeatedly
☐	☒	The statistical test(s) used AND whether they are one- or two-sided<br>*Only common tests should be described solely by name; describe more complex techniques in the Methods section.*
☐	☒	A description of all covariates tested
☐	☒	A description of any assumptions or corrections, such as tests of normality and adjustment for multiple comparisons
☐	☒	A full description of the statistical parameters including central tendency (e.g. means) or other basic estimates (e.g. regression coefficient) AND variation (e.g. standard deviation) or associated estimates of uncertainty (e.g. confidence intervals)
☐	☒	For null hypothesis testing, the test statistic (e.g. *F*, *t*, *r*) with confidence intervals, effect sizes, degrees of freedom and *P* value noted<br>*Give P values as exact values whenever suitable.*
☒	☐	For Bayesian analysis, information on the choice of priors and Markov chain Monte Carlo settings
☐	☒	For hierarchical and complex designs, identification of the appropriate level for tests and full reporting of outcomes
☒	☐	Estimates of effect sizes (e.g. Cohen's *d*, Pearson's *r*), indicating how they were calculated

Our web collection on statistics for biologists contains articles on many of the points above.

Software and code

Policy information about availability of computer code

Data collection	No software was used in data collection
Data analysis	Computational analysis of eCLIP data was performed using the Skipper resources available on Github [https://github.com/YeoLab/skipper]. Metascape v3.5 was used for Gene Ontology analysis. The gel analyzer feature in ImageJ v1.53k software was used to quantify western blot and and DNA gel electrophoresis images. JupyterLab 4.0.4 was used for general data analysis, using Python 3.10.11 and the pandas, scipy, matplotlib, seaborn, and pybedtools packages. Genomic alignment of RNA-seq data was performed using STAR 2.7.6a. Differential alternative splicing events were detected using rMATS 4.0.2. biomaRt 2.50.3 running under R 4.1.3 was used to identify genes with specified GO annotations. Differential expression was detected using DeSeq2. Proteomics raw data was analyzed by Spectronaut v16.065 (Biognosys) using a UniProt database (Homo sapiens, UP000005640), and MS/MS searches were performed under BGS factory settings. Spectromine v4.2.230428.52329 was used to analyze proteomics data in follow-up experiments. gRNA sequences were designed using the cas13design tool. InterProScan v89 was used to predict protein domains for subcloning.

For manuscripts utilizing custom algorithms or software that are central to the research but not yet described in published literature, software must be made available to editors and reviewers. We strongly encourage code deposition in a community repository (e.g. GitHub). See the Nature Portfolio guidelines for submitting code & software for further information.

Data

Policy information about availability of data

All manuscripts must include a data availability statement. This statement should provide the following information, where applicable:

- Accession codes, unique identifiers, or web links for publicly available datasets
- A description of any restrictions on data availability
- For clinical datasets or third party data, please ensure that the statement adheres to our policy

RNA-seq and eCLIP-seq data of this study are available at NCBI-GEO (accession code GSE232599). We referenced the UniPort database (Homo sapiens, UP000005640) and GO Term annotations for analysis of proteomics raw data. We referenced the Ensembl BioMart database for determining splicing-associated proteins using GO Term annotation. We referenced the COMPARTMENTS database for subcellular localization scores of candidates. We referenced the SpliceosomeDB database to identify previous experiments that captured our candidates in the spliceosome.

Human research participants

Policy information about studies involving human research participants and Sex and Gender in Research.

Reporting on sex and gender	n/a
Population characteristics	n/a
Recruitment	n/a
Ethics oversight	n/a

Note that full information on the approval of the study protocol must also be provided in the manuscript.

Field-specific reporting

Please select the one below that is the best fit for your research. If you are not sure, read the appropriate sections before making your selection.

☒ Life sciences ☐ Behavioural & social sciences ☐ Ecological, evolutionary & environmental sciences

For a reference copy of the document with all sections, see nature.com/documents/nr-reporting-summary-flat.pdf

Life sciences study design

All studies must disclose on these points even when the disclosure is negative.

Sample size	Sample size for each experiment is indicated in the figure legend for each experiment. The sample size was chosen based on previous experience for each experiment. No statistical methods were used to predetermine sample size. Experiments were performed in triplicate as per previously published high-throughput tethered function assays (Luo, EC., Nathanson, J.L., Tan, F.E. et al. Large-scale tethered function assays identify factors that regulate mRNA stability and translation. Nat Struct Mol Biol 27, 989–1000 (2020). https://doi.org/10.1038/s41594-020-0477-6). RNA-level validation with agarose gels was performed in duplicate due to the experimental burden required and the availability of a visual readout.
Data exclusions	No data were excluded.
Replication	Independent replication was performed for candidates from the screens as described in the text. All reported findings from the screens were successfully replicated.
Randomization	No randomization was employed. All comparisons are made across experiments in HEK293T cells under controlled experimental conditions, therefore random allocation of samples is not relevant.
Blinding	Blinding is not relevant to this study since group allocation does not occur.

Reporting for specific materials, systems and methods

We require information from authors about some types of materials, experimental systems and methods used in many studies. Here, indicate whether each material, system or method listed is relevant to your study. If you are not sure if a list item applies to your research, read the appropriate section before selecting a response.

Materials & experimental systems

n/a	Involved in the study
	☒ Antibodies
	☒ Eukaryotic cell lines
☒	☐ Palaeontology and archaeology
☒	☐ Animals and other organisms
☒	☐ Clinical data
☒	☐ Dual use research of concern

Methods

n/a	Involved in the study
☒	☐ ChIP-seq
☒	☐ Flow cytometry
☒	☐ MRI-based neuroimaging

Antibodies

Antibodies used

Primary Antibodies:

Rabbit V5 Epitope Tag - Bethyl A190-10A, Lot 7, No Clone Listed (used for IP)
Rabbit TRNAU1AP - GeneTex GTX121631, Lot 40863, No Clone Listed (used for IP)
Rabbit PRPF39 - Thermo Fisher PA5-21627, Lot yg3989185b, No Clone Listed (used for Western Blot at 1:1000 dilution)
Rabbit UPF1 - Cell Signaling Technology D15G6, Lot 3, No Clone Listed (used for Western Blot at 1:1000 dilution)
Mouse GAPDH - Millipore MAB374, Lot 3855179, Clone 6C5 (used for Western Blot at 1:10000 dilution)

Secondary Antibodies:
Goat Anti-Rabbit IgG, HRP Linked - Cell Signaling 7074, Lot 29, No Clone Listed (used for Western Blot at 1:2000 dilution)
800CW Goat Anti-Mouse IgG - Licor 926-32210, Lot d2012535, No Clone Listed (used for Western Blot at 1:2000 dilution)

Validation

Extended Data Figure 4 provided with the manuscript shows validation of antibodies used for eCLIP. All antibodies used for Western Blots were validated by the manufacturer. Reactivity for human samples is noted for each antibody on the manufacturer's website. Validation for other antibodies from the manufacturers' websites are noted below.

PA5-21627: PA5-21627 targets PRPF39 in IHC (P) and WB applications and shows reactivity with Human samples. Validated from manufacturer's website and included citations. Validation included blotting for PRPF39 in PRPF39-transfected HEK293T cells. https://www.thermofisher.com/antibody/product/PRPF39-Antibody-Polyclonal/PA5-21627

D15G6: UPF1 (D15G6) Rabbit mAb recognizes endogenous levels of total UPF1 protein. Species Reactivity: Human, Mouse, Rat, Monkey. Validated from manufacturer's website and included citations. Validation included blotting for UPF1 in various cell lines. https://www.cellsignal.com/products/primary-antibodies/upf1-d15g6-rabbit-mab/12040

MAB374: species reactivity: human, feline, pig, mouse, rabbit, fish, canine, rat. Validated from manufacturer's website and included citations. Validation included blotting for GAPDH in various cell lines. https://www.emdmillipore.com/US/en/product/Anti-Glyceraldehyde-3-Phosphate-Dehydrogenase-Antibody-clone-6C5,MM_NF-MAB374

7074: Species Reactivity: Rabbit. Validated from manufacturer's website and included citations. Validated with a variety of CST primary antibodies for western immunoblotting. https://www.cellsignal.com/products/secondary-antibodies/anti-rabbit-igg-hrp-linked-antibody/7074

926-32210: Species Reactivity: Mouse. Validated from manufacturer's website and included citations. Validation includes applications using ApoTrack primary mAb cocktail in Staurosporine treated and non-treated HeLa cells.https://www.licor.com/bio/reagents/irdye-800cw-goat-anti-mouse-igg-secondary-antibody

Eukaryotic cell lines

Policy information about cell lines and Sex and Gender in Research

Cell line source(s)

Lenti-X HEK293T cells were purchased from Takara Bio.

Authentication

Cell lines were not further authenticated.

Mycoplasma contamination

The cell lines used in this study have been routinely and frequently tested negative for mycoplasma contamination.

Commonly misidentified lines
(See ICLAC register)

No cell lines used are listed in the database of commonly misidentified cell lines.
