# Supplementary material for: Large-scale evaluation of the ability of RNA-binding proteins to activate exon inclusion
Source: Nat Biotechnol. 2024 Jan 2;42(9):1429–41. doi: 10.1038/s41587-023-02014-0 (PMC11389820; doi:10.1038/s41587-023-02014-0)

Source Data Extended Data Figure 6

Unprocessed Western Blot from Extended Data Figure 6a. Both scans are from the same membrane.

PRPF39

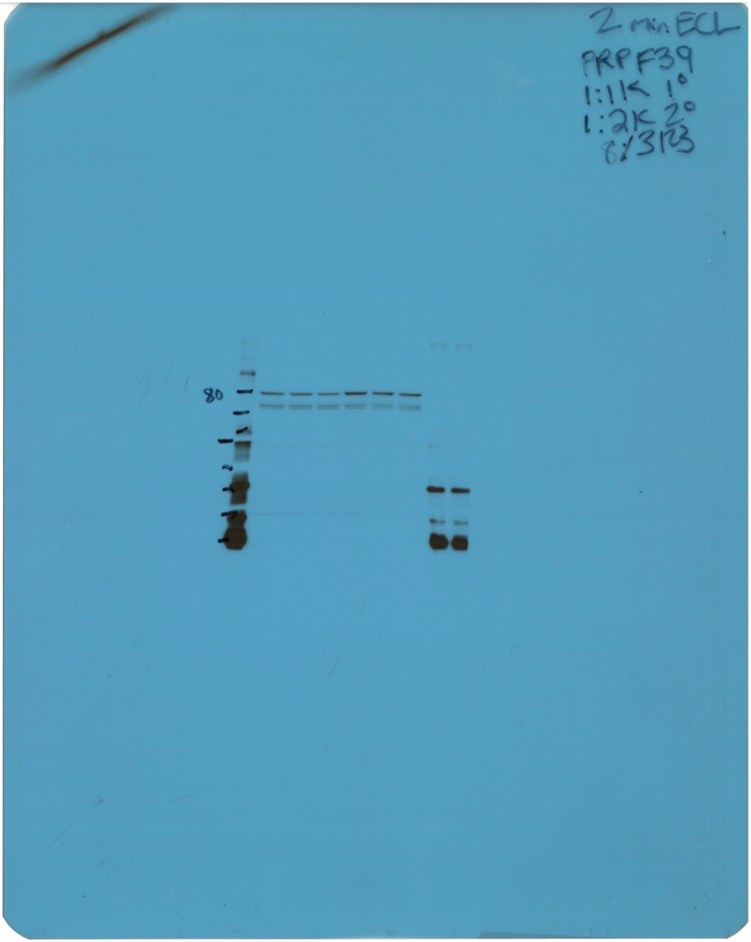

GAPDH

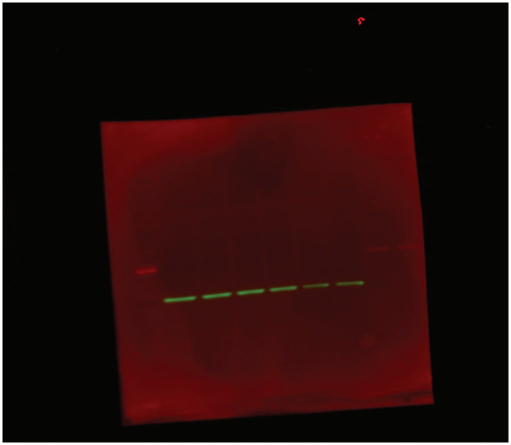

Supplement: Supplementary file 4 — Unprocessed western blot from Extended Data Fig. 6a. Both scans are from the same membrane. [file 41587_2023_2014_MOESM4_ESM.pdf]
